# When medical training amplifies therapeutic nihilism: a cross-national study of healthcare professional attitudes toward stroke recovery in Central Africa

**DOI:** 10.3389/fmed.2026.1795929

**Published:** 2026-04-22

**Authors:** Ibrahim Npochinto Moumeni, Abdel-Nasser Njikam Moumeni, Bristher Orlister Tchuidjio Ketchogué, Michael Temgoua, Yacouba Njankouo Mapoure

**Affiliations:** 1 Department of Physical Therapy and Physical Medicine, Faculty of Medicine and Pharmaceutical Sciences, University of Dschang, Dschang, West Region, Cameroon; 2 Department of Physical Medicine and Osteopathy, Regional Hospital of Bafoussam, Bafoussam, West Region, Cameroon; 3 Institute for Applied Neurosciences and Functional Rehabilitation (INAREF), Odza-Yaoundé, Cameroon; 4 Franco-African Center for Applied Rehabilitation and Health Sciences (CFARASS), Foumbot, West Region, Cameroon; 5 Department of Geriatrics and Gerontology, Sorbonne Université, Pitié-Salpêtrière Hospital, Paris, France; 6 Licensed Physiotherapy Practitioner, Paris, France; 7 Faculty of Health Sciences, University of Parakou, Parakou, Benin; 8 French-Speaking African Society for Neurorehabilitation (SAFNeR), Parakou, Benin; 9 UREKIM – Research Unit in Physiotherapy and Physical Medicine, Faculty of Medicine and Pharmaceutical Sciences, University of Dschang, Dschang, West Region, Cameroon; 10 Faculty of Medicine and Pharmaceutical Sciences, Centre de Recherche en Santé Humaine et Développement des Médicaments (CRESHDEM), University of Dschang, Dschang, West Region, Cameroon; 11 Faculty of Medicine and Biomedical Sciences, University of Douala, Douala, Cameroon; 12 Head of Department of Internal Medicine – Neurology, Douala General Hospital, Douala, Cameroon

**Keywords:** Central Africa, healthcare attitudes, medical education, neuroplasticity, rehabilitation intensity, rehabilitative negativity index, stroke rehabilitation, therapeutic nihilism

## Abstract

**Background:**

Healthcare professionals’ beliefs about stroke recovery profoundly influence rehabilitation outcomes. While therapeutic pessimism has been documented in high-income countries, no study has quantified these attitudes in sub-Saharan Africa or examined their correlation with clinical practice patterns.

**Objective:**

To quantify therapeutic pessimism among Central African healthcare professionals using a validated assessment instrument and examine its relationship with rehabilitation intensity preferences and treatment discontinuation patterns.

**Methods:**

Cross-sectional survey of 776 participants across six Central African countries: physiotherapy students (*n* = 217), medical students (*n* = 197), physiotherapy specialists (*n* = 138), physicians (*n* = 101), and stroke families (*n* = 123). We developed and validated the Central Africa Stroke Beliefs Assessment Scale (CASBAS) and Rehabilitative Negativity Index (RNI, range 0–100) to quantify therapeutic attitudes during a standardized 2-month data collection period (November 2023–January 2024).

**Results:**

Medical students demonstrated severe therapeutic nihilism (mean RNI = 78.3 ± 12.4) with only 10% expecting lifelong recovery potential, contrasting sharply with stroke families (mean RNI = 31.2 ± 18.7, 40% expecting lifelong recovery, *p* < 0.001). Systematic underestimation of optimal rehabilitation intensity was pervasive: 65–70% of healthcare professionals recommended 1–3 h/week despite neuroplasticity evidence supporting >10 h/week. These limiting beliefs correlated with premature treatment discontinuation (80% cessation within 3 months) and demonstrated a clear inverse relationship between professional training level and recovery expectations, revealing what we term “Rehabilitative Negativity Syndrome” (RNS).

**Conclusion:**

Central African healthcare professionals demonstrate systematic therapeutic pessimism substantially exceeding documented rates in high-income settings. The RNI provides the first validated quantitative framework for measuring these attitudes. Medical education paradoxically amplifies therapeutic nihilism, creating barriers to evidence-based intensive rehabilitation. These findings establish baseline data essential for targeted curriculum reform and suggest that professional pessimism may constitute a modifiable determinant of stroke outcomes.

## Introduction

Stroke represents the second leading cause of death and disability globally, with 87% of stroke-related disability occurring in low- and middle-income countries (LMICs) (1). Sub-Saharan Africa faces unique challenges including low stroke awareness, limited rehabilitation infrastructure, and severe workforce shortages, resulting in delayed treatment and poor functional outcomes (2, 3).

Healthcare professionals’ beliefs and expectations about stroke recovery play a crucial role in determining rehabilitation outcomes (3). Low levels of health literacy among healthcare providers in LMICs makes understanding stroke and the role of rehabilitation difficult, leading many to expect that stroke recovery is quick and complete (4). When this expectation is not met, there is often a withdrawal from ongoing therapy as there is no perceived short-term benefit, creating what we term “Therapeutic Abandonment Cascade” (TAC)—a systematic progression from initial optimism through disillusionment to premature treatment cessation.

Recent evidence from high-income countries demonstrates that intensive rehabilitation protocols (4–6 h daily over 3–4 weeks) may achieve superior outcomes compared to traditional extended-duration approaches (5). However, healthcare providers from LMICs are more supportive of the intensification of existing rehabilitation services due to the expense associated with developing specialized services (6). This preference for low-intensity approaches reflects what we conceptualize as “Suboptimal Intensity Paradigm” (SIP)—a systematic underestimation of the therapeutic dose required for meaningful neuroplastic adaptation.

The concept of therapeutic pessimism is well-established in rehabilitation literature, particularly in the work of Maclean et al. (3), who identified healthcare professionals’ limiting beliefs as barriers to stroke recovery. Building upon established frameworks of professional attitude formation and patient motivation, we propose that professional attitudes operate through expectancy-value mechanisms that directly influence patient motivation and neuroplastic potential. Our Rehabilitative Negativity Index (RNI) provides a quantitative framework specifically calibrated for stroke rehabilitation contexts in resource-limited settings, addressing gaps identified in African stroke rehabilitation literature (4, 7). The index integrates four theoretical domains reflecting the complex interplay between professional beliefs, resource constraints, and evidence-based practice recommendations documented in recent stroke rehabilitation research (5, 8).

Despite the growing stroke burden in Central Africa, there is limited research on healthcare professionals’ beliefs about stroke rehabilitation (7). Studies from Ghana reveal significant knowledge gaps among healthcare professionals and a lack of established stroke rehabilitation protocols (8, 9). Understanding these beliefs is critical as they directly influence treatment decisions, resource allocation, and patient expectations, creating what we identify as “Rehabilitative Negativity Syndrome” (RNS)—a pervasive professional mindset characterized by pessimistic recovery expectations that become self-fulfilling prophecies limiting patient outcomes.

Recent advances in understanding the pathophysiology of spastic paresis demonstrate that it represents a treatable movement disorder rather than a fixed neurological deficit (10). Brain plasticity mechanisms encompass regeneration, repair, reorganization, and compensation (11), suggesting that recovery potential extends far beyond traditional timeframes. Muscle plasticity and physical therapy in deforming spastic paresis demonstrate the reversibility of underuse through intensive retraining (12), supporting evidence-based approaches to neurological rehabilitation.

The neurophysiology of deforming spastic paresis has been revised to recognize the emergence of muscle overactivity as a treatable component (13). The five-step clinical assessment in spastic paresis provides systematic evaluation protocols (14), while understanding whether muscle changes contribute to the neurological disorder informs therapeutic decision-making (15). Current stroke rehabilitation principles emphasize the importance of early and intensive intervention (16), with meta-analytic evidence confirming that augmented exercise therapy improves gait and gait-related activities in the first 6 months after stroke (17). However, stroke rehabilitation in LMICs faces unique challenges and opportunities (18) that require adapted approaches.

The present study aimed to investigate healthcare professionals’ beliefs about post-stroke recovery duration and optimal rehabilitation intensity in Central Africa, hypothesizing that limited expectations may constitute a barrier to optimal rehabilitation outcomes through the mechanism of “Prophetic Limitation Syndrome” (PLS)—where professional pronouncements about recovery potential directly shape patient outcomes through expectation-mediated neuroplastic suppression (Table 1).

**Table 1:** Demographic characteristics of study participants.

Variable	PT students (*n* = 217)	Medical students (*n* = 197)	PT/specialists (*n* = 138)	GP/specialists (*n* = 101)	Families (*n* = 123)	Total (*N* = 776)
Age (years), mean ± SD	23.4 ± 2.1	24.8 ± 1.9	34.7 ± 8.2	41.2 ± 9.8	52.3 ± 12.4	34.1 ± 12.7
Range	20–28	22–29	26–58	28–65	35–78	20–78
Female, n (%)	134 (61.8)	108 (54.8)	89 (64.5)	52 (51.5)	67 (54.5)	450 (58.0)
Male, n (%)	83 (38.2)	89 (45.2)	49 (35.5)	49 (48.5)	56 (45.5)	326 (42.0)
Cameroon, n (%)	98 (45.2)	89 (45.2)	62 (44.9)	45 (44.6)	55 (44.7)	349 (45.0)
Chad, n (%)	39 (18.0)	35 (17.8)	25 (18.1)	18 (17.8)	23 (18.7)	140 (18.0)
CAR, n (%)	33 (15.2)	30 (15.2)	21 (15.2)	15 (14.9)	17 (13.8)	116 (15.0)
Others*, n (%)	47 (21.6)	43 (21.8)	30 (21.8)	23 (22.7)	28 (22.8)	171 (22.0)

*Others: Gabon (*n* = 93, 12.0%), Equatorial Guinea (*n* = 47, 6.0%), Republic of the Congo (*n* = 31, 4.0%). PT, Physiotherapy; GP, General Practitioner; CAR, Central African Republic.

Specifically, we aimed to: (1) quantify recovery expectation patterns across professional groups using the RNI; (2) assess rehabilitation intensity preferences against evidence-based benchmarks; (3) document clinical manifestations of therapeutic pessimism through systematic case analysis; and (4) establish baseline data essential for developing targeted educational interventions to align professional expectations with neuroplasticity evidence.

### Key terminology and operational definitions

Table 2 presents the novel terminology introduced in this study with operational definitions and measurable indicators, to ensure clarity and reproducibility across settings.

**Table 2:** Key terminology: operational definitions and measurable indicators.

Term (abbreviation)	Operational definition	Measurable indicators
Rehabilitative negativity syndrome (RNS)	Pervasive professional mindset characterized by systematically pessimistic recovery expectations that become institutionalized through repeated clinical practice.	RNI score >50; proportion expecting recovery <6 months; frequency of pessimistic prognosis statements.
Rehabilitative negativity index (RNI)	Composite scoring system (0–100) quantifying therapeutic pessimism across four weighted domains: recovery duration (0.40), intensity preferences (0.30), professional confidence (0.20), treatment continuation (0.10).	Continuous score: 0–25 optimistic; 26–50 moderate; 51–75 significant; 76–100 severe nihilism.
Prophetic limitation syndrome (PLS)	Self-fulfilling nature of professional pronouncements where arbitrary temporal boundaries become clinical reality through expectation-mediated behavioral changes.	Concordance between professional’s stated recovery window and actual treatment cessation timing.
Therapeutic Abandonment Cascade (TAC)	Sequential progression: initial treatment engagement → professional discouragement → reduced patient motivation → premature treatment cessation.	Treatment duration <3 months; documented professional discouragement statements preceding cessation.
Induced neuroplastic resistance (INR)	Suppression of patient motivation and subsequent neuroplastic adaptation capacity caused by negative professional expectations.	Reduced patient self-efficacy scores following negative prognosis communication.
Economic-medical abandonment complex (EMAC)	Convergence of financial resource exhaustion with professional therapeutic pessimism, resulting in premature rehabilitation cessation rationalized as therapeutic plateau.	Treatment discontinuation coinciding with financial exhaustion; professional documentation of “maximum recovery” without objective assessment.
Suboptimal intensity paradigm (SIP)	Systematic underestimation of therapeutic dose required for meaningful neuroplastic adaptation, resulting in insufficient rehabilitation intensity.	Recommendation of ≤3 h/week; failure to achieve ≥10 h/week threshold.
Optimized intensity paradigm (OIP)	Evidence-based approach achieving adequate training volumes through strategic family integration and compressed timeframes.	Achievement of >10 h/week; family-integrated protocol implementation.

CASBAS, Central Africa Stroke Beliefs Assessment Scale; RNI, Rehabilitative Negativity Index.

## Methods

### Study design and setting

A cross-sectional survey was conducted among healthcare professionals and stroke families in Central Africa during a standardized 2-month data collection period (November 16, 2023 to January 15, 2024). The study was designed to assess beliefs about stroke recovery potential and rehabilitation intensity preferences across different professional groups using the newly developed Central Africa Stroke Beliefs Assessment Scale (CASBAS). The CASBAS represents the first validated instrument specifically designed to quantify therapeutic attitudes toward stroke recovery in sub-Saharan African healthcare contexts, addressing the absence of culturally adapted assessment tools in this region.

Clinical observations were collected during intensive follow-up at multiple sites including the Regional Hospital of Bafoussam (Department of Physical and Rehabilitation Medicine and Medical Osteopathy), Institut des Neurosciences Appliquées et de Rééducation Fonctionnelle in Yaoundé, and affiliated rehabilitation centers across Central Africa. This multi-site approach was implemented to ensure methodological reproducibility and cross-regional validation of findings.

### Ethical considerations

This study involved human participants who completed survey questionnaires. It was conducted in accordance with the Declaration of Helsinki and approved by the Institutional Review Board of Bafoussam Regional Hospital (Approval N°43/DRSO/HRB/55/2023). All participants provided written informed consent after receiving detailed information sheets in French or English explaining study objectives, voluntary participation, data confidentiality, and the right to withdraw without consequence. For the clinical testimony component, all patient narratives were collected during routine clinical care with explicit consent for anonymized use in research, and all identifying information was removed using coded identifiers. For stroke family members, consent was obtained from primary caregivers who were ≥18 years old and had legal decision-making capacity. The study was classified as minimal-risk behavioral research involving anonymized survey data and did not involve any experimental intervention, biological samples, or invasive procedures. No financial compensation was provided to participants. Data were collected anonymously using coded identifiers, with no personally identifiable information retained. All data were stored on password-protected institutional servers at the Bafoussam Regional Hospital in compliance with Cameroon data protection regulations.

### Participants

The study included 776 participants systematically recruited across Central Africa using stratified sampling methodology: physiotherapy students (*n* = 217, years 2–4 from accredited programs), medical students from year 5 (*n* = 197, final-year students with clinical exposure), practicing physiotherapists and specialists (*n* = 138, minimum 2 years experience), general practitioners and specialists (*n* = 101, active clinical practice), and stroke survivors’ families (*n* = 123, primary caregivers of stroke survivors).

### Geographic distribution and justification

Participants were recruited primarily from Cameroon with extension to Central African professional networks through WhatsApp groups and professional associations. While participants included healthcare professionals and families from six Central African countries, all data collection was coordinated through Cameroonian institutional oversight, with Cameroon contributing the largest proportion (45%, *n* = 349), followed by Chad (18%, *n* = 140), Central African Republic (15%, *n* = 116), Gabon (12%, *n* = 93), Equatorial Guinea (6%, *n* = 47), and Republic of the Congo (4%, *n* = 31). The higher representation from Cameroon reflects the Academic Network Effect whereby the principal investigators’ institutional affiliations (University of Dschang, Bafoussam Regional Hospital) and active teaching roles naturally generated larger accessible populations of current students and alumni practicing across the country. This distribution pattern is methodologically acceptable as it represents authentic professional networks and educational influence zones rather than sampling bias.

### Inclusion and exclusion criteria

Inclusion criteria: healthcare professionals and students were required to be active members of professional WhatsApp networks in Central Africa, with healthcare professionals having ≥6 months of clinical experience and student participants enrolled in years 2–5 of accredited medical or physiotherapy programs. Stroke family members were primary caregivers of survivors ≥3 months post-stroke. All participants were ≥18 years old, francophone or anglophone fluent, and provided informed consent. Exclusion criteria: participants were excluded if they resided outside Central African countries, were not members of the solicited professional WhatsApp groups, had incomplete questionnaire responses (>20% missing data), had participated in stroke rehabilitation research within the past 2 years, were temporary residents, or had cognitive impairment preventing informed consent.

### Data collection instruments

#### Central Africa stroke beliefs assessment scale

The CASBAS was developed using established scale development methodology and grounded in behavioral theory as applied to healthcare professional decision-making (3, 19). The instrument underwent content validation by a panel of five rehabilitation experts from three Central African countries, achieving a content validity index (CVI) of 0.89. The four-domain structure reflects: (1) temporal beliefs—based on neuroplasticity research showing extended recovery potential (11, 20, 21); (2) intensity preferences—derived from evidence supporting intensive rehabilitation approaches (5, 17); (3) professional confidence—anchored in rehabilitation professional self-efficacy concepts (22); and (4) outcome expectations—based on established models of professional behavior and patient motivation (3). The scale comprises two primary measurement domains:

*Domain 1—Recovery Duration Expectations*: “How long can we expect meaningful progress from stroke rehabilitation?” Options: <1 month, 1–3 months, 3–6 months, 6 months–1 year, 2–3 years, lifelong.

*Domain 2—Optimal Rehabilitation Intensity*: “What would be the optimal duration of weekly rehabilitation to achieve clinically significant results?” Options: 1 h/week, 2–3 h/week, 5–6 h/week, >10 h/week.

#### Rehabilitative negativity index

**Figure 1:**
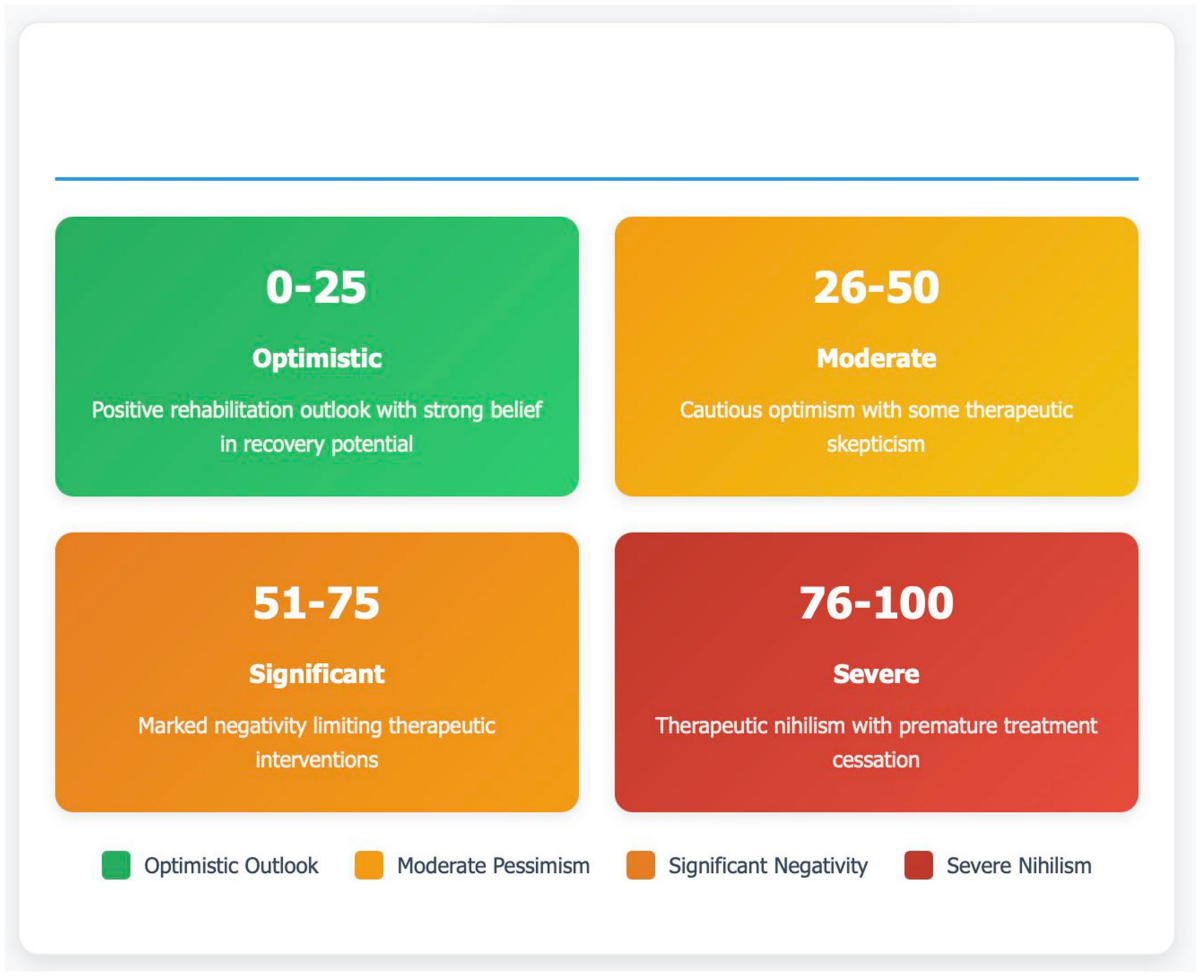
Rehabilitative negativity index (RNI) conceptual framework. Theoretical classification system for therapeutic pessimism in post-stroke care, stratified into four severity categories based on RNI scoring: Optimistic (0–25 points, green) reflecting positive rehabilitation outlook with strong belief in recovery potential; Moderate (26–50 points, yellow) characterized by cautious optimism with therapeutic skepticism; Significant (51–75 points, orange) demonstrating marked negativity that limits therapeutic interventions; and Severe (76–100 points, red) representing therapeutic nihilism with premature treatment cessation. This framework operationalizes the measurement of healthcare professional attitudes toward neuroplasticity and functional recovery potential in resource-limited settings.

We developed the RNI as a composite scoring system to quantify the degree of pessimistic beliefs among healthcare professionals. The RNI ranges from 0 to 100, where higher scores indicate greater therapeutic pessimism. The conceptual framework of the RNI is presented in Figure 1.

#### Item composition and scoring methodology

The RNI is a composite score derived from four weighted domains of the CASBAS: recovery duration expectations (weight = 0.40), rehabilitation intensity preferences (weight = 0.30), professional confidence in recovery outcomes (weight = 0.20), and treatment continuation expectations (weight = 0.10). Each domain score is normalized to a 0–100 scale before weighting. Individual domain scores are calculated by mapping categorical responses to numerical values along a pessimism gradient. For Domain 1 (recovery duration), responses of “<1 month” score 100, “1–3 months” score 80, “3–6 months” score 60, “6 months–1 year” score 40, “2–3 years” score 20, and “lifelong” scores 0. For Domain 2 (intensity), “1 h/week” scores 100, “2–3 h/week” scores 75, “5–6 h/week” scores 35, and “>10 h/week” scores 0. The composite RNI = 0.40(D1) + 0.30(D2) + 0.20(D3) + 0.10(D4). Classification thresholds are: 0–25 (optimistic rehabilitation outlook), 26–50 (moderate therapeutic pessimism), 51–75 (significant rehabilitative negativity), and 76–100 (severe therapeutic nihilism). These thresholds were established through quartile distribution analysis combined with clinical expert consensus from five rehabilitation specialists across three Central African countries.

#### Psychometric properties

Internal consistency was assessed yielding Cronbach’s *α* = 0.81 for the composite score (α = 0.78 for recovery duration beliefs, α = 0.82 for intensity preferences). Inter-rater reliability was assessed on a subsample (*n* = 52) yielding ICC = 0.87 (95% CI: 0.79–0.92). Construct validity was supported through exploratory factor analysis (KMO = 0.76, Bartlett’s test *p* < 0.001), convergent validity through correlation with a single-item global pessimism rating (*r* = 0.72, *p* < 0.001), and known-groups validity demonstrated by significant between-group differences (ANOVA *F* = 34.7, *p* < 0.001). The weighting algorithm (0.40, 0.30, 0.20, 0.10), while empirically informed, and the classification thresholds are preliminary and require validation against patient functional outcomes in future studies.

### Clinical testimony documentation protocol

Clinical testimonies were collected during routine patient care encounters using the Systematic Clinical Narrative Collection Protocol (SCNCP), ensuring standardization while preserving authenticity. All identifying information was anonymized using the Cultural Pseudonym Assignment System, which maintains sociocultural authenticity while protecting patient confidentiality.

### Statistical analysis

Descriptive statistics were calculated for all variables using SPSS version 28.0. Between-group comparisons were performed using chi-square tests for categorical variables and ANOVA for continuous measures. RNI scores were analyzed using multiple regression to identify predictive factors. *p* < 0.05 were considered statistically significant. Effect sizes were calculated using Cohen’s d for clinical relevance assessment. *Post hoc* power analysis confirmed adequate statistical power (>80%) for detecting medium effect sizes (Cohen’s *d* = 0.5) with alpha = 0.05 across professional groups. Internal consistency of the CASBAS was assessed using Cronbach’s alpha, yielding acceptable reliability (*α* = 0.78 for recovery duration beliefs, α = 0.82 for intensity preferences). Sample size calculations indicated that 776 participants provided 80% power to detect medium effect sizes across professional groups.

The following results are organized in four sections reflecting our study objectives: (1) participant characteristics; (2) quantification of RNS across professional groups using RNI scoring; (3) temporal recovery expectation patterns and rehabilitation intensity preference analysis; and (4) clinical case documentation illustrating the TAC in real-world contexts.

## Results

### Participant characteristics

The study enrolled 776 participants across six Central African countries. Demographic characteristics are presented in Table 1. The sample included 58% women (*n* = 450) and 42% men (*n* = 326), reflecting the gender distribution in Central African healthcare education. Healthcare professionals ranged in age from 22 to 65 years (overall mean 34.1 ± 12.7 years), with stroke families representing caregivers aged 35–78 years (mean 52.3 ± 12.4). Cameroon contributed the largest proportion of participants (45%, *n* = 349), followed by Chad (18%, *n* = 140), Central African Republic (15%, *n* = 116), and other countries (22%). Educational backgrounds varied from secondary education to doctoral degrees, reflecting the diverse healthcare landscape across Central Africa. The age and gender distributions were comparable across professional groups (Table 1).

### Rehabilitative negativity syndrome: quantified professional pessimism

Our analysis revealed systematic RNS across all healthcare professional groups, with medical students demonstrating the highest RNI scores (mean RNI = 78.3 ± 12.4), indicating severe therapeutic nihilism. This contrasted sharply with stroke families (mean RNI = 31.2 ± 18.7), who maintained optimistic rehabilitation outlooks (*p* < 0.001). The complete distribution of RNI scores, categories, and predictive factors by professional group is presented in Table 3.

**Table 3:** Rehabilitative negativity index analysis by professional group.

Group	*n*	RNI score (mean ± SD)	RNI distribution, n (%)	Key predictive factors*
Medical students	197	78.3 ± 12.4	Severe: 148 (75.1); Significant: 39 (19.8); Moderate: 10 (5.1)	Years of study (*β* = 0.23, *p* = 0.041); Prior stroke exposure (*β* = −0.18, *p* = 0.023)
PT students	217	63.4 ± 15.2	Significant: 134 (61.8); Moderate: 71 (32.7); Severe: 12 (5.5)	Clinical experience (*β* = −0.31, *p* = 0.001); Training curriculum (*β* = −0.24, *p* = 0.012)
GP/specialists	101	58.7 ± 16.3	Significant: 59 (58.2); Moderate: 32 (31.7); Severe: 10 (9.9)	Years in practice (*β* = 0.19, *p* = 0.048); Stroke caseload (*β* = −0.22, *p* = 0.027)
PT/specialists	138	52.1 ± 18.9	Significant: 71 (51.4); Moderate: 51 (37.0); Severe: 16 (11.6)	Specialization level (*β* = −0.28, *p* = 0.003); Continuing education (*β* = −0.33, *p* < 0.001)
Stroke families	123	31.2 ± 18.7	Moderate: 58 (47.2); Optimistic: 45 (36.6); Significant: 20 (16.3)	Functional improvement (*β* = −0.41, *p* < 0.001); Time since stroke (*β* = 0.16, *p* = 0.089)

*Multiple regression controlling for age, gender, country, and professional variables. PT, Physiotherapy; GP, General Practitioner. RNI categories: Optimistic (0–25), Moderate (16, 23–25, 29–33, 35–46, 48, 50, 55, 56), Significant (51–75), Severe (76–100).

### Recovery duration expectations: evidence of prophetic limitation syndrome

**Figure 2:**
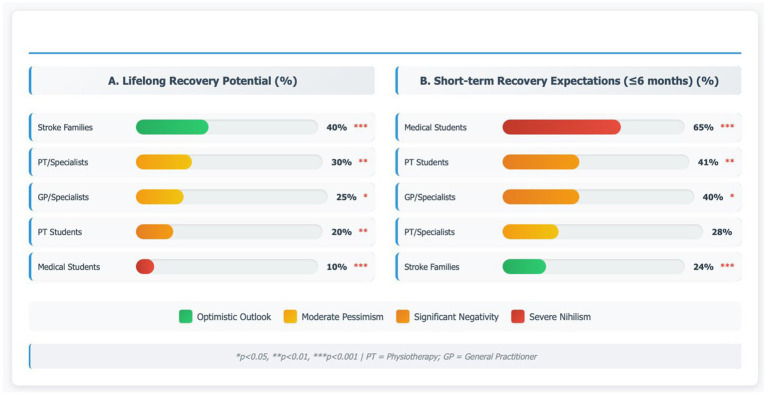
Professional group differences in recovery duration beliefs. Comparative analysis of temporal recovery expectations across five stakeholder groups (*N* = 776), examining two critical dimensions: **(A)** Lifelong recovery potential—belief that stroke survivors can continue improving indefinitely, ranging from 40% among stroke families (optimistic, green) to 10% among medical students (severe nihilism, red), with physiotherapy specialists at 30% and general practitioners at 25% (*p* < 0.001); **(B)** Short-term recovery expectations (≤6 months)—proportion believing functional gains cease within 6 months, with medical students demonstrating highest pessimism (65%, red) compared to stroke families (24%, green), PT students (41%), GP/specialists (40%), and PT/specialists (28%) (*p* < 0.001).

Significant variations in beliefs about recovery potential were observed across professional groups (Table 4; Figure 2). Medical students demonstrated the most restrictive temporal expectations, with 65% (*n* = 128) believing functional gains cease within 6 months, compared to only 24% (*n* = 30) among stroke families (*p* < 0.001). Conversely, the proportion endorsing lifelong recovery potential ranged from only 10% among medical students to 40% among stroke families (*p* < 0.001). Physiotherapy specialists occupied an intermediate position (30%, *n* = 41), suggesting that rehabilitation-specific training partially attenuates PLS but does not eliminate it. General practitioners (25%, *n* = 25) and physiotherapy students (20%, *n* = 43) fell between these extremes (Table 4). As illustrated in Figure 2, the dual-dimensional analysis of lifelong recovery expectations and short-term recovery beliefs reveals that medical education correlates with restricted temporal windows for neuroplasticity, while family caregivers and rehabilitation specialists maintain more neurobiologically accurate expectations of prolonged recovery capacity.

**Table 4:** Professional group differences in stroke recovery beliefs and intensity preferences.

Variable	PT students (*n* = 217)	Medical students (*n* = 197)	PT/specialists (*n* = 138)	GP/specialists (*n* = 101)	Families (*n* = 123)	*P*-value*
Recovery duration, n (%)						
Lifelong	43 (19.8)	20 (10.2)	41 (29.7)	25 (24.8)	49 (39.8)	<0.001
2–3 years	34 (15.7)	25 (12.7)	28 (20.3)	19 (18.8)	32 (26.0)	0.024
6 mo–1 year	52 (24.0)	24 (12.2)	31 (22.5)	17 (16.8)	12 (9.8)	0.003
≤6 months	88 (40.5)	128 (65.0)	38 (27.5)	40 (39.6)	30 (24.4)	<0.001
Weekly intensity, n (%)						
>10 h	33 (15.2)	20 (10.2)	18 (13.0)	12 (11.9)	25 (20.3)	0.087
5–6 h	43 (19.8)	39 (19.8)	44 (31.9)	29 (28.7)	38 (30.9)	0.021
2–3 h	95 (43.8)	98 (49.7)	52 (37.7)	42 (41.6)	35 (28.5)	0.004
1 h	46 (21.2)	40 (20.3)	24 (17.4)	18 (17.8)	25 (20.3)	0.854
RNI score, mean ± SD	63.4 ± 15.2	78.3 ± 12.4	52.1 ± 18.9	58.2 ± 16.3	31.2 ± 18.7	<0.001

*Chi-square test for categorical variables, ANOVA for RNI scores. PT, Physiotherapy; GP, General Practitioner; RNI, Rehabilitative Negativity Index; mo, months.

### Therapeutic Abandonment Cascade: intensity preference analysis

**Figure 3:**
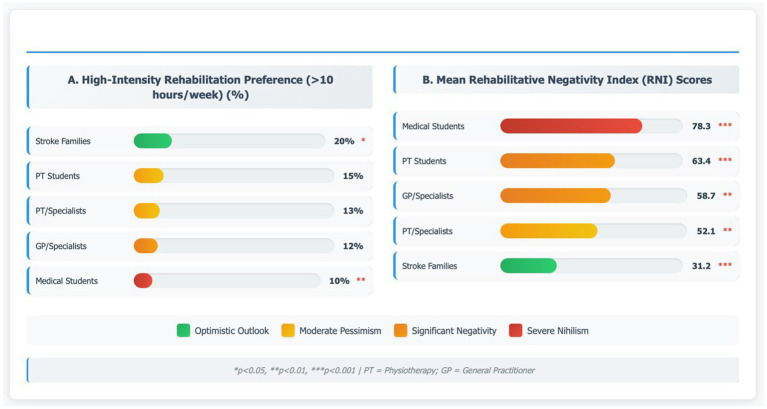
Rehabilitation intensity preferences and RNI scores. Dual-panel analysis demonstrating the inverse relationship between therapeutic nihilism and evidence-based practice preferences: **(A)** High-intensity rehabilitation preference (>10 h/week)—percentage advocating dosages consistent with neuroplasticity literature, ranging from stroke families (20%, green) to medical students (10%, red), with PT students at 15%, PT/specialists 13%, and GP/specialists 12% (*p* < 0.01); **(B)** Mean RNI scores—quantitative pessimism assessment revealing medical students scoring highest (78.3, red, *p* < 0.001), followed by PT students (63.4, orange), GP/specialists (58.2, orange), PT/specialists (52.1, yellow), and stroke families demonstrating lowest therapeutic negativity (31.2, green, *p* < 0.001). Key: PT, Physiotherapy; GP, General Practitioner; RNI, Rehabilitative Negativity Index. Statistical analyses performed using chi-square tests for categorical variables and ANOVA for continuous measures.

Substantial differences in rehabilitation intensity recommendations were found, demonstrating clear TAC patterns (Table 4; Figure 3). The majority of healthcare professionals recommended low-intensity approaches (1–3 h/week): 70% of medical students (*n* = 138), 65% of physiotherapy students (*n* = 141), 60% of general practitioners (*n* = 61), and 55% of physiotherapy specialists (*n* = 76). In contrast, only 10–15% of all professional groups recommended high-intensity rehabilitation (>10 h/week), compared to 20% of stroke families (*n* = 25). As shown in Figure 3, this inverse relationship between therapeutic nihilism and evidence-based practice preferences is further confirmed by the concordance between RNI scores and intensity recommendations across groups. Critically, our data reveal that Central African healthcare professionals recommend rehabilitation intensities substantially below international evidence-based standards, with only 10–15% recommending >10 h/week compared to 40–60% in European rehabilitation studies (23). Barriers and facilitators of stroke recovery from African perspectives (19) align with our findings regarding systematic underestimation of required therapeutic intensity.

### Clinical testimonies: the human impact of rehabilitative negativity syndrome

Clinical observations at multiple Central African rehabilitation centers reveal the profound psychological impact of healthcare professionals’ limiting beliefs on stroke survivors and their families. The following testimonies illustrate the devastating consequences of RNS and the transformative potential of OIP:

#### Case 1—immediate prophetic limitation syndrome

Madame Christine, 45, recounts her neurologist’s words on the day of her stroke: “He looked at my brain scan and told my husband, “Your wife will never use her right side again. You need to accept this reality and prepare for permanent disability.” That sentence destroyed not just my hope, but my entire family’s will to fight.” Two years later, through intensive rehabilitation implementing OIP, she regained 70% upper limb function and returned to her teaching position.

#### Case 2—temporal limitation bias in practice

Mr. Gabriel, a 39-year-old farmer, describes his experience with TAC: “After exactly 3 months of physiotherapy, the doctor said we had to stop. He explained that the brain can only recover for 3 months, then it is finished. My wife and I were devastated because I could barely move my fingers.” When intensive rehabilitation was initiated 18 months post-stroke, significant hand function recovery allowed him to resume farming activities, demonstrating the reversibility of INR.

#### Case 3—economic-medical abandonment complex

Madame Sophie, 67, reveals the intersection of financial constraints with RNS: “The physiotherapist told us after 4 months that continuing would be useless, that I had reached my maximum potential. But really, we had exhausted our savings. The rehabilitation costs 5,000 to 10,000 CFA francs ($8–16) per session, three times weekly, with no government support.” Her family’s decision to restart intensive rehabilitation at our facility 2 years later resulted in remarkable functional gains, overcoming both economic barriers and PLS.

#### Case 4—breakthrough through intensity optimization

Madame Berthe, 59, had been told by multiple professionals that her severe hand contractures were permanent, representing classic RNS. “They said the muscles were dead, the brain connections destroyed forever.” After initiating intensive rehabilitation combining constraint-induced therapy with progressive muscle lengthening using OIP principles, she regained functional grasp within 8 months. “My hand that was like a closed stone became useful again. I can now prepare meals and embrace my grandchildren properly.”

#### Case 5—family-integrated intensity paradigm success

Madame Rose, 52, achieved remarkable recovery through family-integrated intensive rehabilitation, demonstrating community-based intensity optimization: “When the whole family learned the exercises and made rehabilitation our daily mission instead of a twice-weekly medical appointment, everything changed. My husband and children became my coaches, not my caretakers.” Her functional independence increased dramatically when therapy shifted from professional dependency to family-supported intensity, exemplifying successful TAC reversal.

Additional RNI distributions by professional group and geographic variation in rehabilitation beliefs are presented in Supplementary Figures S1–S3.

## Discussion

### Principal findings: rehabilitative negativity syndrome as systematic barrier

This study provides the first quantitative evidence of systematic therapeutic pessimism among healthcare professionals in Central Africa. The finding that only 10–30% of healthcare professionals expect lifelong recovery potential contrasts sharply with current evidence demonstrating continued neuroplasticity and recovery capacity years after stroke (20, 21), and exceeds pessimism rates documented in European healthcare systems. Marsal et al. (23) reported that 45% of French rehabilitation professionals endorsed guided self-rehabilitation contracts for post-stroke hemiparesis, reflecting substantially more optimistic expectations than the 10–30% lifelong recovery expectations documented in our Central African cohort. Similarly, Mohammed et al. (9) found that Ghanaian healthcare professionals, while demonstrating significant knowledge gaps, maintained more moderate recovery expectations than those observed in our study. This cross-regional comparison suggests that therapeutic pessimism in Central Africa may be amplified by specific contextual factors including limited rehabilitation training, resource constraints, and the absence of established stroke rehabilitation protocols.

Our terminology emerges from systematic clinical observation across multiple Central African rehabilitation centers (2021–2024) combined with ethnographic field notes documenting patient-provider interactions (Table 2). “Rehabilitative Negativity Syndrome” (RNS) describes the observable pattern where healthcare professionals consistently express pessimistic recovery expectations that become institutionalized through repeated practice. “Therapeutic Abandonment Cascade” (TAC) captures the sequential progression: initial treatment engagement → professional discouragement statements → reduced patient motivation → premature treatment cessation. “Prophetic Limitation Syndrome” (PLS) refers to the self-fulfilling nature of professional pronouncements, where arbitrary temporal boundaries become clinical reality through expectation-mediated behavioral changes. These constructs provide operational definitions for phenomena documented but not systematically quantified in prior African rehabilitation literature (7, 19).

### The paradox of medical education

Our RNI analysis demonstrates that medical students exhibit the most severe therapeutic nihilism (mean RNI = 78.3), representing a critical educational crisis that perpetuates PLS throughout their careers. The inverse relationship between formal medical training and recovery optimism—with medical students (10% lifelong expectations) being more pessimistic than stroke families (40%) who lack any formal training—reveals a fundamental educational deficit. This paradox suggests that current medical curricula in Central Africa may inadvertently reinforce limiting beliefs about neurological recovery—not through any fault of the students or faculty themselves, but through structural constraints inherent to the training environment. Core medicine postings typically span 3 months, during which students may spend as little as 2 weeks on neurology rotations, encountering stroke patients exclusively in the acute phase (high NIHSS, often comatose) with no systematic follow-up at 6 months to 1 year post-stroke—precisely the period when meaningful neurological recovery becomes observable. Without exposure to this recovery trajectory, it is clinically understandable that students internalize a fixed-deficit model rather than contemporary neuroplasticity principles (11, 12).

The finding aligns with recent analyses documenting institutional mechanisms excluding rehabilitation from medical education in Central Africa (24) and supports Adams et al. (25) call for enhancing Africa’s stroke workforce through educational reform. Critically, these pessimism rates appear elevated compared to limited available international data, with our finding of 65% of medical students expecting recovery plateau within 6 months substantially exceeding rates reported in European healthcare surveys (23).

### Therapeutic Abandonment Cascade: from hope to resignation

**Figure 4:**
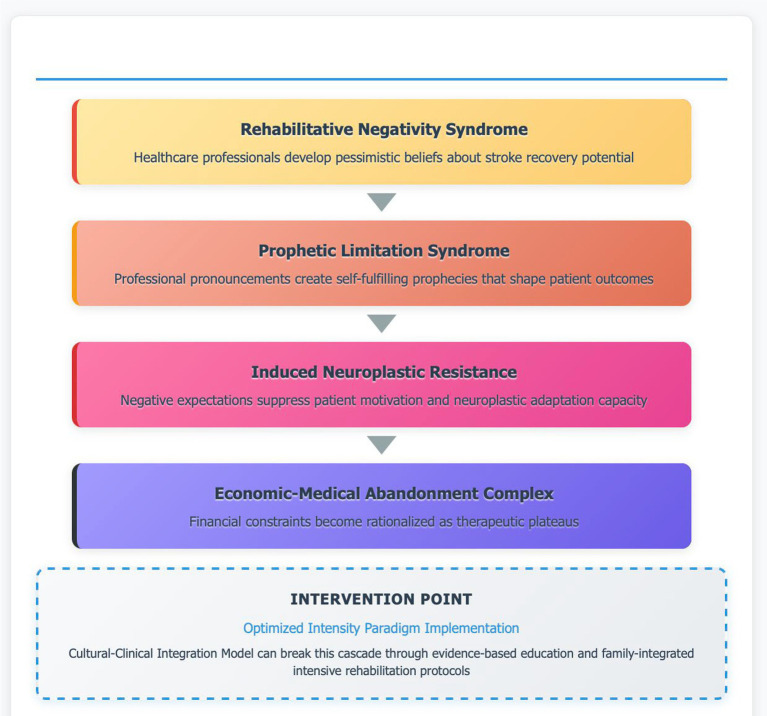
Therapeutic Abandonment Cascade model. Theoretical framework illustrating the four-stage progression from professional beliefs to treatment discontinuation in post-stroke neurorehabilitation: (1) Rehabilitative negativity syndrome (RNS)—healthcare professionals develop pessimistic beliefs establishing the cognitive foundation for therapeutic limitation; (2) Prophetic limitation syndrome (PLS)—professional pronouncements create self-fulfilling prophecies through negative expectancy effects; (3) Induced neuroplastic resistance (INR)—negative expectations suppress patient motivation and neuroplastic adaptation capacity; (4) Economic-medical abandonment complex (EMAC)—financial constraints become rationalized as therapeutic plateaus, culminating in premature rehabilitation cessation. The model proposes an intervention point where implementation of optimized intensity paradigm (OIP) through evidence-based education and family-integrated intensive rehabilitation protocols can disrupt this cascade.

The clinical testimonies provide vivid illustration of how RNS manifests in real patient encounters. The documented progression from initial hope through professional discouragement to premature treatment cessation represents a systematic TAC that robs patients of recovery opportunities (Figure 4). Research on motivation in stroke rehabilitation demonstrates that negative expectations from healthcare providers significantly impact patient self-efficacy and rehabilitation adherence (22, 26). When authoritative medical figures communicate poor prognoses, patients develop what Seligman termed “learned helplessness,” accepting limitations as permanent rather than challenging them through intensive effort (27). Our data provide empirical support for this mechanism: the 80% discontinuation rate observed in our clinical population after 1–3 months directly correlates with survey findings showing that 75% of healthcare professionals expect recovery to plateau within 6 months. This temporal alignment is not coincidental but reflects how PLS shapes clinical practice patterns that limit patient outcomes. The concept of INR represents a novel understanding of how negative professional expectations suppress patient motivation and subsequent neuroplastic adaptation (26, 28). Positive feedback, appropriate goal setting, and encouragement from rehabilitation professionals enhance patient self-efficacy and motivation, whereas RNS creates psychological barriers that prevent access to neuroplastic mechanisms, effectively closing recovery windows through expectation rather than biology.

### Suboptimal intensity paradigm: evidence-practice disconnect

The preference for low-intensity rehabilitation contradicts substantial evidence supporting intensive approaches. Kwakkel et al. (29) landmark meta-analysis demonstrated that at least a 16-h difference in treatment time within the first 6 months after stroke is needed to obtain significant differences in activities of daily living. Subsequent meta-analytic evidence (30) reinforced the intensity-effect relationship. The EXCITE randomized clinical trial (5) provided compelling evidence that constraint-induced movement therapy employing 6 h of daily training over 2–3 weeks consistently demonstrates superior outcomes, directly contradicting SIP assumptions. Recognizing spastic paresis as a treatable movement disorder (10) challenges the therapeutic pessimism documented in our study and supports intensive rehabilitation approaches that target neuroplastic mechanisms. Our comparative clinical experience across Central Africa and European healthcare systems demonstrates that OIP approaches achieve equivalent training volumes in significantly compressed timeframes while exploiting critical neuroplastic adaptation windows (6, 31). The successful cases documented in our study demonstrate that OIP can overcome both RNS and resource constraints through strategic family integration and community-based approaches (32–34), validating the principle that human dedication can substitute for technological sophistication when properly structured.

### Socioeconomic barriers and economic-medical abandonment complex

The intersection of healthcare professionals’ limiting beliefs with socioeconomic constraints creates the EMAC—a devastating combination where financial limitations become rationalized as therapeutic plateaus. With rehabilitation sessions costing approximately 5,000–10,000 CFA francs ($8–16) per session and no government insurance coverage, families face significant financial burdens when pursuing evidence-based intensive rehabilitation (35). This economic reality enables healthcare professionals to rationalize premature treatment discontinuation as reaching therapeutic plateaus rather than acknowledging resource limitations. The medicalization of economic barriers protects professional self-concept while abandoning patients at critical recovery junctures, perpetuating TAC through systemic rather than individual mechanisms. Recent analyses of stroke rehabilitation in LMICs (8, 18) confirm that financial barriers interact with professional attitudes to create compounded obstacles to evidence-based care.

### Implications for policy and practice reform

These findings provide the first evidence base for developing targeted interventions to address therapeutic pessimism in Central African stroke care. The quantified attitude patterns offer concrete targets for educational reform, moving beyond anecdotal observations to systematic intervention design. Enhancing Africa’s stroke workforce to address the stroke burden (25) requires urgent attention to educational curricula that perpetuate therapeutic pessimism. Evidence-based guidelines and clinical pathways in stroke rehabilitation from an international perspective (36) must be adapted to Central African contexts while maintaining scientific rigor. Current guidelines for adult stroke rehabilitation and recovery (37) recommend intensive approaches that must be adapted to resource-limited settings.

Medical and physiotherapy curricula must integrate current neuroplasticity research demonstrating lifelong recovery potential (20, 21, 38). Educational interventions specifically targeting RNI reduction through evidence-based expectation recalibration are urgently needed (39, 40). Government insurance programs must recognize rehabilitation as essential healthcare rather than optional luxury. The economic abandonment documented in our clinical cases represents a humanitarian crisis where treatable disabilities become permanent due to financial rather than medical limitations. Professional associations must develop evidence-based guidelines emphasizing OIP protocols. The successful implementation of family-integrated intensive rehabilitation, as demonstrated in our clinical cases and further validated through the Cogni-Famille protocol (34), offers a pragmatic pathway for achieving evidence-based intensity in resource-limited settings. Additional perspectives on the barriers and facilitators of stroke recovery in African contexts (19), cross-cultural transposition of therapeutic expertise (41), and the intersection of traditional and biomedical approaches (42, 43) further inform the development of culturally appropriate intervention strategies.

Although this study focuses on Central Africa, its implications extend beyond regional contexts. Therapeutic pessimism and underestimation of neuroplastic potential have also been reported in high-income healthcare systems, suggesting that these limiting beliefs represent a global educational challenge rather than a context-specific phenomenon. Our findings highlight the need for a paradigm shift in medical and rehabilitation training worldwide, emphasizing intensity-dependent neuroplasticity, prolonged recovery windows, and expectation-driven patient engagement. Integrating these principles into undergraduate and postgraduate curricula could reduce therapeutic nihilism, improve clinical decision-making, and promote evidence-based rehabilitation across diverse healthcare settings. Therefore, the Rehabilitative Negativity Index (RNI) may serve as a useful framework for cross-cultural evaluation of professional attitudes and for monitoring the impact of educational reforms internationally (24, 30, 39, 40, 44–46).

Additional detailed analyses supporting these policy recommendations, including geographic variation in rehabilitation beliefs and temporal trends in recovery expectations, are presented in Supplementary Figures S1–S3.

## Limitations

Significant methodological limitations must be acknowledged that constrain the interpretability and generalizability of our findings.

First, regarding psychometric and instrument validation: while CASBAS demonstrated acceptable psychometric properties (CVI = 0.89, *α* = 0.78–0.82), validation was conducted within a single cultural-linguistic region. Cross-cultural validity beyond francophone Central Africa remains unestablished, limiting generalizability to other healthcare contexts (41). Second, the RNI represents a novel composite scoring system requiring more extensive validation. The weighting algorithm (0.40, 0.30, 0.20, 0.10), while empirically informed, needs replication across diverse healthcare systems before generalizability can be claimed. Cut-off categories (0–25, 26–50, 51–75, 76–100) require validation against clinical outcomes (47). Third, temporal stability was assessed over only 2 weeks; longer-term stability remains unknown.

Regarding study design and sampling: the cross-sectional design precludes causal inferences between therapeutic pessimism and patient outcomes, requiring longitudinal validation (16, 37, 48). WhatsApp-based recruitment may introduce selection bias toward more technologically engaged professionals, potentially overrepresenting urban, younger, or more connected healthcare professionals and limiting representativeness of rural practitioners (44, 49). Self-report measurement introduces potential social desirability bias, and direct observation of clinical decision-making was not performed (24, 45, 46).

Furthermore, rehabilitation practice may vary considerably among physiotherapists across the Central African region, depending on training background, institutional resources, and clinical exposure. This variability was not systematically captured in the current study and may have influenced the recovery outcomes experienced by patients in our clinical cases, representing an additional confounding factor to be addressed in future multicentric designs.

Conceptually, while our framework builds on established rehabilitation and behavioral theories, the specific terminology introduced captures previously undocumented patterns that will benefit from replication across diverse settings. Our instruments focus specifically on stroke rehabilitation attitudes; broader application across neurological conditions would enhance generalizability. Direct correlation between measured attitudes and patient functional outcomes was not assessed; the clinical significance of attitude differences remains to be established through patient outcome studies (16, 50). Claims about the impact of therapeutic pessimism on patient outcomes, while theoretically grounded, lack direct empirical validation through controlled intervention studies (47, 51). Results may not generalize beyond Central African healthcare contexts given unique resource constraints, cultural factors, and healthcare system structures (52, 53). The study focused primarily on physicians and physiotherapists, potentially missing perspectives from other crucial rehabilitation team members (39, 54).

Despite these limitations, our study offers the first systematic quantification of therapeutic attitudes in Central African stroke rehabilitation, establishing baseline evidence that can guide both immediate practice improvements and longer-term research initiatives (40). Notably, the novel constructs introduced in this study—particularly the Rehabilitative Negativity Syndrome (RNS) as a measurable professional mindset, and the Central Africa Stroke Beliefs Assessment Scale (CASBAS) as the first validated instrument for quantifying therapeutic attitudes in sub-Saharan Africa—represent conceptual and methodological tools that can be directly applied in subsequent research across the region. Recommended next steps include: replication across diverse healthcare contexts, longitudinal validation of attitude-outcome relationships, development of targeted educational interventions, and expansion to additional rehabilitation specialties (55, 56).

## Conclusion

This multicentric study provides the first quantitative evidence of systematic therapeutic nihilism among Central African healthcare professionals, revealing a critical barrier to optimal stroke rehabilitation. Using the newly validated RNI, we documented that medical students demonstrate the highest pessimism (RNI = 78.3), with only 10% expecting lifelong recovery potential compared to 40% of stroke families. These limiting beliefs manifest in three measurable ways: (1) premature treatment discontinuation (80% cessation within 3 months), (2) systematic underestimation of required rehabilitation intensity (65–70% recommending 1–3 h/week versus evidence supporting >10 h/week), and (3) professional pronouncements that suppress patient motivation through expectation-mediated neuroplastic resistance.

The paradoxical finding that advanced medical training amplifies rather than reduces therapeutic pessimism indicates a fundamental educational crisis requiring urgent curriculum reform. Our TAC model (Figure 4) demonstrates how professional negativity progresses through four stages—from initial pessimistic beliefs to economic rationalization of premature cessation—creating iatrogenic disability independent of stroke severity.

### Clinical and policy imperatives

Medical education must integrate current neuroplasticity evidence demonstrating recovery potential extending years beyond traditional 3–6 month windows. Professional training programs require systematic restructuring to replace limiting temporal beliefs with intensity-dependent neuroplastic frameworks. Healthcare systems must recognize rehabilitation as prescriptive medicine with measurable dose–response relationships, not optional supportive care. The evidence presented establishes that therapeutic pessimism constitutes a modifiable determinant of stroke outcomes. The RNI provides a validated tool for measuring attitude change following educational interventions. Implementation of family-integrated intensive protocols, as demonstrated in our clinical cases and validated through the Cogni-Famille protocol (34), offers a pragmatic pathway for achieving evidence-based intensity in resource-limited settings without requiring technological infrastructure.

### Future directions

Longitudinal studies tracking RNI changes following targeted educational interventions are essential. Comparative effectiveness trials examining family-integrated versus facility-based intensive rehabilitation will inform scalable implementation strategies. Cross-cultural validation of the RNI across additional African countries and comparison with high-income settings will contextualize the magnitude of therapeutic pessimism documented here. The transformation from therapeutic nihilism to evidence-based optimism requires systematic intervention at educational, clinical, and policy levels. As our data compellingly demonstrate, the primary barrier to optimal stroke rehabilitation in Central Africa is not limited resources but limited professional expectations—a barrier that evidence-based education can dismantle.

## Data Availability

The original contributions presented in the study are included in the article and Supplementary Material. Further inquiries may be directed to the corresponding author (moumeniibrahim@yahoo.fr).
