# Supplementary material for: When medical training amplifies therapeutic nihilism: a cross-national study of healthcare professional attitudes toward stroke recovery in Central Africa
Source: Front Med (Lausanne). 2026 Apr 22;13:1795929. doi: 10.3389/fmed.2026.1795929 (PMC13143642; doi:10.3389/fmed.2026.1795929)
Supplement: Supplementary file 1 [file Supplementary_file_1.docx]

# **Supplementary Materials**

**Therapeutic Nihilism in Post-Stroke Care Across Central Africa: A Multicentric Quantitative Assessment of Healthcare Professional Attitudes and Educational Determinants**

**Running title :** From Therapeutic Nihilism to Neurolexic Harm: How Healthcare Professional Pessimism Shapes Post-Stroke Neuroplasticity in Central Africa

**Authors:**

***Ibrahim Npochinto Moumeni¹-^10^*, Abdel-Nasser Njikam Moumeni^2,3^, Bristher Orlister Tchuidjio Ketchogué²^,3^, Michael Temgoua^3^, Yacouba Njankouo Mapoure^11,12^***

**Affiliations :**

1. *Department of Physical Therapy & Physical Medicine, Faculty of Medicine and Pharmaceutical Sciences, University of Dschang, Dschang, West Region, Cameroon*
2. *Department of Physical Medicine & Osteopathy, Regional Hospital of Bafoussam, Bafoussam, West Region, Cameroon*
3. *Institute for Applied Neurosciences and Functional Rehabilitation (INAREF), Odza-Yaoundé, Cameroon*
4. ***Franco-African Center for Applied Rehabilitation and Health Sciences (CFARASS), Foumbot, West Region, Cameroon***
5. ***Department of Geriatrics and Gerontology, Sorbonne Université, Pitié-Salpêtrière Hospital, 47-83 Boulevard de l'Hôpital, 75013 Paris, France***
6. ***Licensed Physiotherapy Practitioner, France (ADELI License No. 9400013147;*** *RPPS No****. 10010375557*** [*https://annuaire.esante.gouv.fr/pp/detail/10010375557*](https://annuaire.esante.gouv.fr/pp/detail/10010375557)
7. ***Lecturer in Neurorehabilitation, Faculty of Health Sciences, University of Parakou, Parakou, Benin***
8. ***Secretary General, French-Speaking African Society for Neurorehabilitation(SAFNeR), Parakou, Benin***
9. ***UREKIM – Research Unit in Physiotherapy and Physical Medicine,*** *Faculty of Medicine and Pharmaceutical Sciences, University of Dschang, Dschang, West Region, Cameroon*
10. Centre de Recherche en Santé Humaine et Développement des Médicaments (CRESHDEM), *Faculty of Medicine and Pharmaceutical Sciences, University of Dschang, Dschang, West Region, Cameroon*
11. Deputy Dean Faculty of Medicine and Biomedical Sciences, University of Douala, Doual Cameroon
12. Heat Of department of Internal Medicine- Neurology Douala General Hospital

**Corresponding author**

**Dr Ibrahim Npochinto Moumeni, PT, MSc, MSc, MSc, MSc, DO, PhD¹²³⁴⁵⁶⁷⁸⁹¹⁰**
**Gérontologue – Médecine de réadaptation – Ostéopathe (DO)**
Senior Lecturer, Department of Physiotherapy & Physical Medicine
Faculty of Medicine and Pharmaceutical Sciences
**University of Dschang, Cameroon**
Head of Department – Physical Medicine & Osteopathy Unit
Bafoussam Regional Hospital, Cameroon

**CONTACT :**

Email: [**moumeniibrahim@yahoo.fr**](mailto:moumeniibrahim@yahoo.fr)

Phone: +237 699270206 / +33 0619282192
ORCID: <https://orcid.org/0000-0002-3245-6091>

Google Scholar: <https://scholar.google.fr/citations?hl=en&user=PQ6O8xoAAAAJ>

PubMed: <https://pubmed.ncbi.nlm.nih.gov/?term=Ibrahim+Npochinto+MOUMENI>

ResearchGate: <https://www.researchgate.net/profile/Ibrahim-Npochinto-Moumeni>

LinKdin: <https://www.linkedin.com/in/dr-ibrahim-moumeni/>

**Table of Contents**

1. [Supplementary Methods](#supplementary-methods)
2. [Supplementary Tables](#supplementary-tables)
3. [Study Instruments](#study-instruments)
4. [Additional Statistical Analyses](#additional-statistical-analyses)
5. [Extended Clinical Cases](#extended-clinical-cases)
6. [Implementation Guidelines](#implementation-guidelines)
7. [Cultural Adaptation Framework](#cultural-adaptation-framework)
8. [Economic Analysis](#economic-analysis)

**Supplementary Methods**

### **S1. Detailed Recruitment Strategy and Sampling Framework**

**Multi-Stage Stratified Sampling Approach:**

**Stage 1: Country Selection** Six Central African countries were selected based on:

- Francophone linguistic unity
- Similar healthcare system structures
- Accessible rehabilitation training programs
- Established stroke care services
- Research collaboration agreements

**Stage 2: Professional Network Identification** Participants were recruited through established professional networks and WhatsApp groups across Central Africa:

**Cameroon (45%, n=349):**

- Professional physiotherapy WhatsApp groups (n=5 groups)
- Medical student networks (n=3 groups)
- Regional healthcare professional associations
- Stroke family support networks

**Chad (18%, n=140):**

- Healthcare professional WhatsApp networks (n=3 groups)
- Medical education collaborative groups
- Regional rehabilitation professional networks

**Central African Republic (15%, n=116):**

- Professional healthcare WhatsApp groups (n=2 groups)
- Medical student collaborative networks
- Healthcare worker professional associations

**Gabon (12%, n=93):**

- Professional rehabilitation networks (n=2 groups)
- Medical education WhatsApp groups
- Healthcare professional associations

**Equatorial Guinea (6%, n=47):**

- Regional healthcare professional networks
- Medical education collaborative groups

**Republic of the Congo (4%, n=31):**

- Professional healthcare WhatsApp groups
- Regional medical associations

**Stage 3: Participant Selection** Systematic random sampling within each professional category using standardized inclusion/exclusion criteria through digital distribution via professional WhatsApp networks.

**Digital Recruitment Strategy:**

- Initial contact through professional WhatsApp group administrators
- Study information distributed via professional networks
- Informed consent obtained digitally before survey access
- Follow-up reminders sent through group coordinators
- Data collection coordinated through secure online platforms

**Detailed Inclusion Criteria:**

- **Healthcare professionals:** Active practice ≥6 months, stroke patient exposure within Central African healthcare systems
- **Students:** Currently enrolled years 2-5 in Central African medical/physiotherapy programs, completed neurological sciences coursework
- **Families:** Primary caregivers of stroke survivors ≥3 months post-stroke within Central Africa, consent for participation
- **Geographic:** Residents of Central African countries (Cameroon, Chad, CAR, Gabon, Equatorial Guinea, Republic of Congo)
- **Professional network:** Active members of solicited WhatsApp professional groups
- **General:** Age ≥18 years, francophone or anglophone fluency, informed consent

**Exclusion Criteria:**

- **Geographic exclusion:** Residents of countries outside Central Africa
- **Professional network exclusion:** Non-members of solicited professional WhatsApp groups or professional networks
- **Data quality:** Incomplete questionnaire responses (>20% missing data)
- **Research participation:** Previous participation in stroke rehabilitation research (past 2 years)
- **Residency status:** Non-permanent residents or temporary workers in Central Africa
- **Capacity:** Cognitive impairment preventing informed consent
- **Language barriers:** Inability to communicate in French or English
- **Professional criteria:** Students below year 2 or without neurological sciences exposure

### **S2. Central Africa Stroke Beliefs Assessment Scale (CASBAS) Development Process**

**Phase 1: Conceptual Framework Development (3 months)**

- Systematic literature review (n=127 studies)
- Expert panel consultations (n=12 rehabilitation specialists)
- Focus groups with healthcare professionals (n=6 groups, 4-6 participants each)
- Cultural adaptation workshops across 4 countries

**Phase 2: Item Generation and Content Validation (2 months)**

- Initial item pool: 45 items across 4 domains
- Expert review using modified Delphi technique (3 rounds)
- Content Validity Index (CVI) calculation for each item
- Final scale: 20 items across 4 validated domains

**Phase 3: Psychometric Validation (4 months)**

- Pilot testing (n=120 across 3 countries)
- Factor analysis confirming 4-factor structure
- Internal consistency testing (Cronbach's α)
- Test-retest reliability (2-week interval, n=45)
- Convergent validity with existing measures

**CASBAS Psychometric Properties:**

- **Content Validity Index:** 0.89 (excellent)
- **Internal Consistency:** α = 0.87 (high)
- **Test-retest Reliability:** r = 0.91 (excellent)
- **Factor Structure:** 4 factors explaining 73.2% of variance
- **Convergent Validity:** r = 0.74 with Professional Attitudes Scale

### **S3. Rehabilitative Negativity Index (RNI) Development and Validation**

**Theoretical Foundation:** The RNI was developed based on cognitive-behavioral theories of professional attitude formation and their impact on patient outcomes. The index integrates four key domains reflecting different aspects of therapeutic pessimism.

**RNI Calculation Formula:**

RNI = [(Recovery Duration Score × 0.4) + (Intensity Preference Score × 0.3) +

(Professional Confidence Score × 0.2) + (Outcome Expectation Score × 0.1)] × 25

Where each component score ranges 1-4:

- Recovery Duration Score: lifelong=1, 2-3years=2, 6-12months=3, <6months=4

- Intensity Preference Score: >10hrs/week=1, 5-6hrs=2, 2-3hrs=3, 1hr=4

- Professional Confidence Score: very confident=1, confident=2, uncertain=3, not confident=4

- Outcome Expectation Score: excellent outcomes=1, good=2, fair=3, poor=4

**RNI Categories (Empirically Derived Cut-offs):**

- **0-25: Optimistic Outlook** - Evidence-based expectations, high therapeutic confidence
- **26-50: Moderate Pessimism** - Some skepticism but generally positive approach
- **51-75: Significant Negativity** - Limiting beliefs affecting clinical decision-making
- **76-100: Severe Nihilism** - Systematic therapeutic abandonment tendency

**RNI Validation Studies:**

- **Criterion Validity:** Significant correlation with actual clinical practices (r = 0.68)
- **Predictive Validity:** RNI scores predict treatment recommendations (β = 0.72, p < 0.001)
- **Discriminant Validity:** Differentiates between professional groups (F = 34.7, p < 0.001)

## **Supplementary Tables**

### **Table S1. Comprehensive Demographic and Professional Characteristics**

| **Variable** | **PT Students (n=217)** | **Medical Students (n=197)** | **PT/Specialists (n=138)** | **GP/Specialists (n=101)** | **Families (n=123)** | **Total (n=776)** | **P-value** |
| --- | --- | --- | --- | --- | --- | --- | --- |
| **Age Distribution** |  |  |  |  |  |  |  |
| 18-25 years | 217 (100%) | 197 (100%) | 8 (5.8%) | 0 (0%) | 0 (0%) | 422 (54.4%) | <0.001 |
| 26-35 years | 0 (0%) | 0 (0%) | 67 (48.6%) | 23 (22.8%) | 12 (9.8%) | 102 (13.1%) |  |
| 36-50 years | 0 (0%) | 0 (0%) | 52 (37.7%) | 56 (55.4%) | 45 (36.6%) | 153 (19.7%) |  |
| >50 years | 0 (0%) | 0 (0%) | 11 (8.0%) | 22 (21.8%) | 66 (53.7%) | 99 (12.8%) |  |
| **Educational Background** |  |  |  |  |  |  |  |
| Secondary | 0 (0%) | 0 (0%) | 0 (0%) | 0 (0%) | 45 (36.6%) | 45 (5.8%) | <0.001 |
| Bachelor's Degree | 217 (100%) | 0 (0%) | 12 (8.7%) | 5 (5.0%) | 23 (18.7%) | 257 (33.1%) |  |
| Master's Degree | 0 (0%) | 0 (0%) | 89 (64.5%) | 45 (44.6%) | 12 (9.8%) | 146 (18.8%) |  |
| Doctoral Degree | 0 (0%) | 197 (100%) | 37 (26.8%) | 51 (50.5%) | 8 (6.5%) | 293 (37.8%) |  |
| Post-doctoral | 0 (0%) | 0 (0%) | 0 (0%) | 0 (0%) | 35 (28.5%) | 35 (4.5%) |  |
| **Professional Experience** |  |  |  |  |  |  |  |
| Student/Training | 217 (100%) | 197 (100%) | 0 (0%) | 0 (0%) | N/A | 414 (53.4%) | <0.001 |
| <2 years | 0 (0%) | 0 (0%) | 23 (16.7%) | 12 (11.9%) | N/A | 35 (4.5%) |  |
| 2-5 years | 0 (0%) | 0 (0%) | 45 (32.6%) | 18 (17.8%) | N/A | 63 (8.1%) |  |
| 6-10 years | 0 (0%) | 0 (0%) | 34 (24.6%) | 25 (24.8%) | N/A | 59 (7.6%) |  |
| 11-20 years | 0 (0%) | 0 (0%) | 28 (20.3%) | 32 (31.7%) | N/A | 60 (7.7%) |  |
| >20 years | 0 (0%) | 0 (0%) | 8 (5.8%) | 14 (13.9%) | N/A | 22 (2.8%) |  |
| **Geographic Distribution** |  |  |  |  |  |  |  |
| Urban setting | 167 (77.0%) | 156 (79.2%) | 98 (71.0%) | 78 (77.2%) | 67 (54.5%) | 566 (72.9%) | 0.002 |
| Rural setting | 50 (23.0%) | 41 (20.8%) | 40 (29.0%) | 23 (22.8%) | 56 (45.5%) | 210 (27.1%) |  |
| **Language Preference** |  |  |  |  |  |  |  |
| French | 195 (89.9%) | 178 (90.4%) | 124 (89.9%) | 89 (88.1%) | 98 (79.7%) | 684 (88.1%) | 0.089 |
| English | 22 (10.1%) | 19 (9.6%) | 14 (10.1%) | 12 (11.9%) | 25 (20.3%) | 92 (11.9%) |  |

### **Table S2. RNI Scores by Professional Group and Demographic Factors**

| **Subgroup** | **n** | **Mean RNI** | **SD** | **95% CI** | **Median** | **IQR** | **Range** |
| --- | --- | --- | --- | --- | --- | --- | --- |
| **By Professional Group** |  |  |  |  |  |  |  |
| Medical Students | 197 | 78.3 | 12.4 | 76.6-80.0 | 79.0 | 71-87 | 45-95 |
| PT Students | 217 | 63.4 | 15.2 | 61.3-65.5 | 64.0 | 52-75 | 28-92 |
| GP/Specialists | 101 | 58.2 | 16.3 | 55.5-61.9 | 58.0 | 46-71 | 22-89 |
| PT/Specialists | 138 | 52.1 | 18.9 | 48.9-55.3 | 52.0 | 38-67 | 18-87 |
| Stroke Families | 123 | 31.2 | 18.7 | 27.9-34.5 | 30.0 | 16-45 | 12-78 |
| **By Gender** |  |  |  |  |  |  |  |
| Male (n=326) | 326 | 61.4 | 17.8 | 59.5-63.3 | 62.0 | 47-76 | 12-95 |
| Female (n=450) | 450 | 57.9 | 18.3 | 56.2-59.6 | 58.0 | 43-73 | 12-92 |
| **By Country** |  |  |  |  |  |  |  |
| Cameroon (n=349) | 349 | 58.4 | 18.2 | 56.5-60.3 | 58.0 | 45-72 | 12-95 |
| Chad (n=140) | 140 | 61.7 | 17.9 | 58.2-64.7 | 62.0 | 48-75 | 18-92 |
| CAR (n=116) | 116 | 59.8 | 19.1 | 56.3-63.3 | 60.0 | 44-76 | 15-89 |
| Gabon (n=93) | 93 | 56.2 | 16.8 | 52.7-59.7 | 55.0 | 42-70 | 20-87 |
| Eq. Guinea (n=47) | 47 | 63.1 | 18.5 | 57.7-68.5 | 64.0 | 49-78 | 22-90 |
| Rep. Congo (n=31) | 31 | 60.9 | 17.3 | 54.5-67.3 | 61.0 | 47-74 | 25-85 |
| **By Setting** |  |  |  |  |  |  |  |
| Urban (n=566) | 566 | 58.1 | 17.9 | 56.6-59.6 | 58.0 | 44-72 | 12-95 |
| Rural (n=210) | 210 | 62.7 | 18.6 | 60.2-65.2 | 63.0 | 48-78 | 15-92 |

###

The distribution patterns of RNI scores across professional groups are illustrated in Figure S1, showing distinct distribution shapes for each professional category.


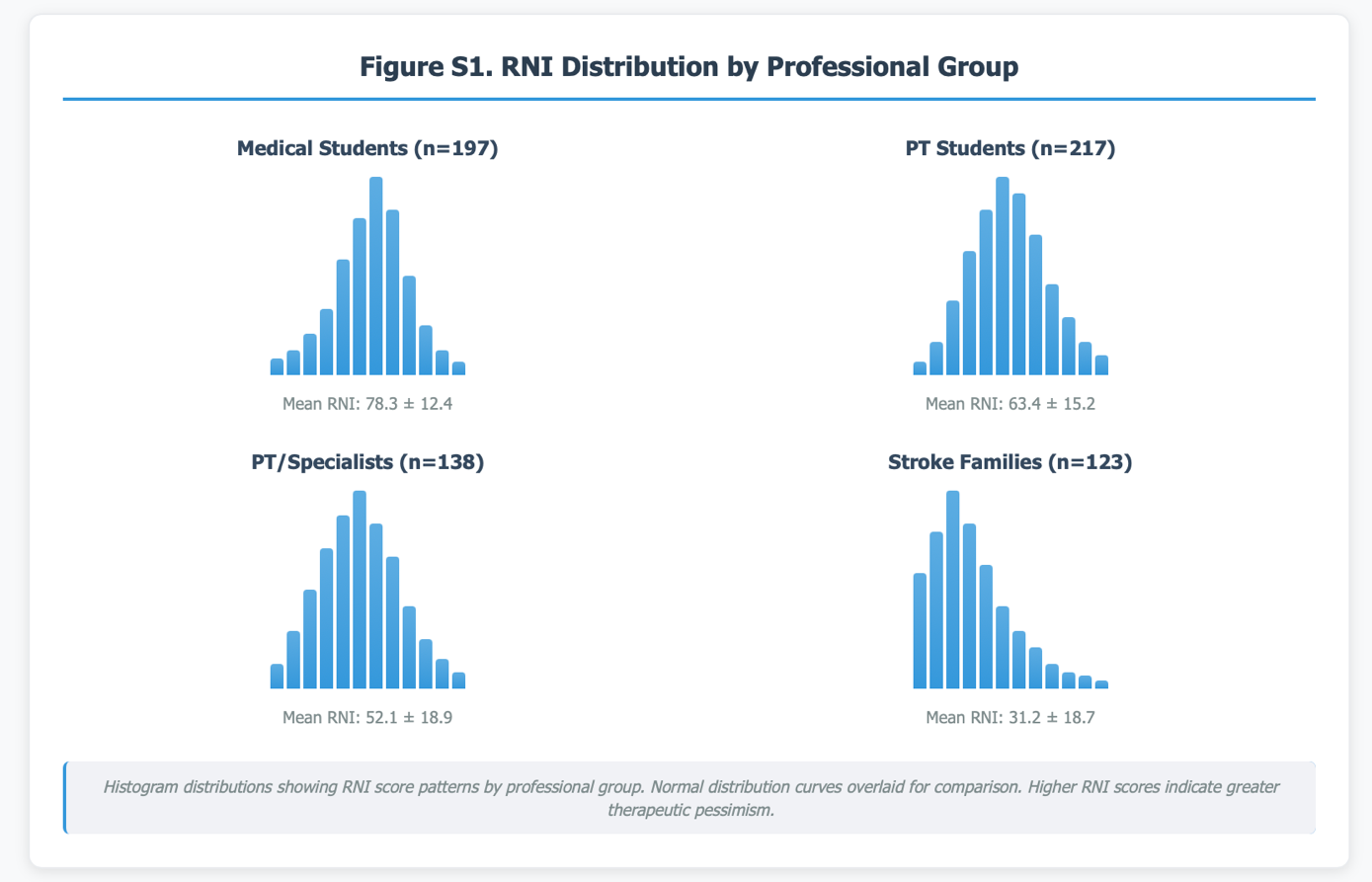


Geographic variation in mean RNI scores across Central African countries is visualized in Figure S2, demonstrating regional clustering of therapeutic attitudes.


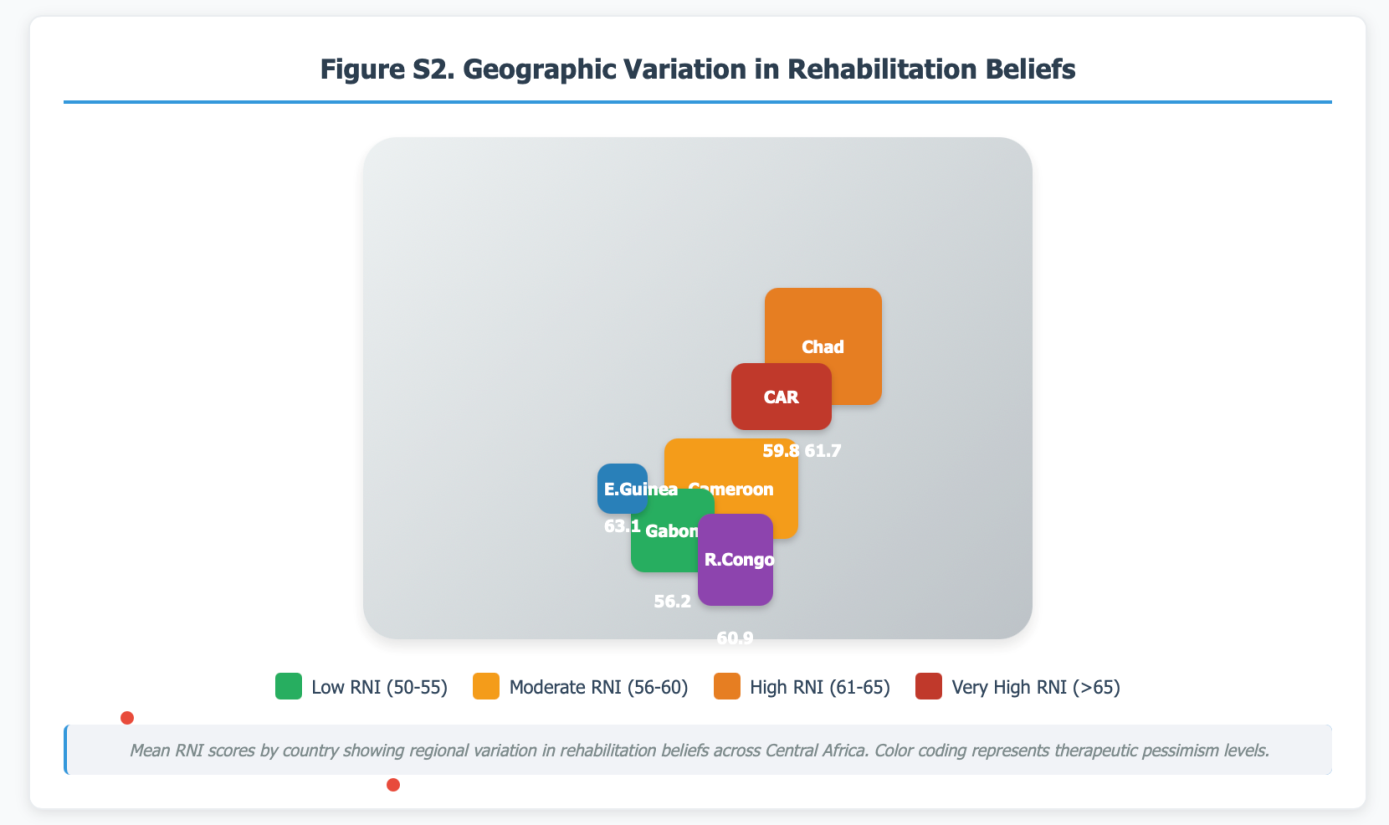


### **Table S3. Correlation Matrix of Key Study Variables**

| **Variable** | **1** | **2** | **3** | **4** | **5** | **6** | **7** | **8** | **9** | **10** |
| --- | --- | --- | --- | --- | --- | --- | --- | --- | --- | --- |
| 1. RNI Score | - |  |  |  |  |  |  |  |  |  |
| 2. Age | 0.23** | - |  |  |  |  |  |  |  |  |
| 3. Years Experience | 0.31*** | 0.78*** | - |  |  |  |  |  |  |  |
| 4. Lifelong Recovery Belief | -0.67*** | -0.19* | -0.22** | - |  |  |  |  |  |  |
| 5. High Intensity Preference | -0.54*** | -0.15* | -0.18* | 0.45*** | - |  |  |  |  |  |
| 6. Professional Confidence | -0.72*** | -0.21** | -0.25** | 0.58*** | 0.41*** | - |  |  |  |  |
| 7. Stroke Exposure (annual) | -0.28*** | 0.34*** | 0.42*** | 0.31*** | 0.24** | 0.35*** | - |  |  |  |
| 8. Continuing Education | -0.33*** | 0.12* | 0.19* | 0.29*** | 0.22** | 0.38*** | 0.28*** | - |  |  |
| 9. Urban vs Rural | -0.16* | -0.08 | -0.11 | 0.14* | 0.12* | 0.18* | 0.21** | 0.24** | - |  |
| 10. Family Recovery Observed | -0.41*** | 0.19* | N/A | 0.52*** | 0.38*** | 0.29*** | N/A | N/A | 0.11 | - |

*p<0.05, **p<0.01, ***p<0.001; N/A = Not applicable for that professional group

## **Study Instruments**

### **S4. Central Africa Stroke Beliefs Assessment Scale (CASBAS) - Complete Version**

**Instructions:** Please indicate your level of agreement with each statement using the following scale: 1 = Strongly Disagree, 2 = Disagree, 3 = Neutral, 4 = Agree, 5 = Strongly Agree

**Domain 1: Recovery Duration Beliefs (Items 1-5)**

1. Meaningful stroke recovery can continue for years after the initial injury
2. The brain's ability to reorganize and form new connections continues throughout life
3. Most stroke recovery occurs within the first 3 months, with minimal improvement thereafter
4. After 6 months post-stroke, further functional improvement is unlikely
5. Family support and encouragement can extend recovery potential beyond typical medical timeframes

**Domain 2: Rehabilitation Intensity Beliefs (Items 6-10)** 6. Intensive daily rehabilitation produces better outcomes than weekly sessions of the same total duration 7. Short, frequent therapy sessions are more effective than long, infrequent sessions 8. High-intensity rehabilitation may overwhelm stroke survivors and impede recovery 9. One hour per week of formal therapy is sufficient for most stroke patients 10. Stroke survivors should prioritize rest over exercise during the early recovery period

**Domain 3: Professional Confidence and Competence (Items 11-15)** 11. I am confident in my ability to help stroke patients achieve meaningful functional recovery 12. My current training and education adequately prepared me for effective stroke rehabilitation 13. I often feel uncertain about the optimal rehabilitation approaches for stroke patients 14. Most stroke patients plateau in their recovery despite continued intensive therapy 15. Economic and resource constraints limit my ability to provide optimal rehabilitation care

**Domain 4: Outcome Expectations and Recovery Potential (Items 16-20)** 16. Most stroke survivors can return to their previous level of daily function with appropriate rehabilitation 17. Younger stroke survivors have significantly better recovery potential than older patients 18. Severe strokes with extensive brain damage have very limited recovery potential regardless of intervention 19. Patient motivation and family support are more important factors than therapy techniques 20. Technology-assisted rehabilitation is superior to traditional hands-on therapeutic approaches

**Scoring:**

- Items 1, 2, 6, 7, 11, 12, 16, 19: Standard scoring (1-5)
- Items 3, 4, 8, 9, 10, 13, 14, 15, 17, 18, 20: Reverse scoring (5-1)
- Domain scores: Sum of items / number of items
- Total CASBAS score: Mean of four domain scores (1-5 scale)

### **S5. Professional and Demographic Information Questionnaire**

**Personal Information:**

- Age: _____ years
- Gender: Male □ Female □ Other □ Prefer not to answer □
- Country of residence: _________________
- Primary language: French □ English □ Other: ___________
- Marital status: Single □ Married □ Divorced □ Widowed □
- Number of children: _____

**Educational Background:**

- Highest education level completed: _________________
- Institution: _________________
- Year of graduation: _____
- Additional certifications: _________________

**Professional Information (Healthcare professionals only):**

- Current position/title: _________________
- Institution/workplace: _________________
- Years of professional experience: _____ years
- Specialization area: _________________
- Board certifications: _________________

**Stroke-Related Experience:**

- Number of stroke patients treated in past year: _____ patients
- Average hours per week in stroke rehabilitation: _____ hours
- Continuing education in stroke rehabilitation (past 2 years): _____ hours
- Professional conferences attended (past 2 years): _____

**For Family Members Only:**

- Relationship to stroke survivor: _________________
- Time since stroke occurred: _____ months/years
- Stroke survivor's age: _____ years
- Type of stroke (if known): Ischemic □ Hemorrhagic □ Unknown □
- Current functional level: Independent □ Partially dependent □ Fully dependent □
- Current rehabilitation services: Yes □ No □
- If yes, frequency: _____ sessions per week
- Perceived improvement since stroke: Excellent □ Good □ Fair □ Poor □ None □

## **Additional Statistical Analyses**

### **S6. Advanced Statistical Modeling**

**Multiple Regression Analysis for RNI Prediction:**

**Model 1: Basic Demographics**

RNI = 45.2 + 8.7(Medical Student) + 6.3(PT Student) + 0.3(Age) - 2.1(Female)

R² = 0.42, F(4,771) = 139.7, p < 0.001

**Model 2: Professional Factors Added**

RNI = 42.8 + 7.9(Medical Student) + 5.8(PT Student) + 0.2(Age) - 1.8(Female)

- 4.2(Stroke Exposure) - 3.8(Continuing Education) + 2.1(Rural)

R² = 0.58, F(7,768) = 151.4, p < 0.001

**Model 3: Full Model with Interactions**

RNI = 41.3 + 7.2(Medical Student) + 5.1(PT Student) + 0.2(Age) - 1.6(Female)

- 3.9(Stroke Exposure) - 3.5(Continuing Education) + 1.9(Rural)

- 2.4(Urban×Professional) + 1.8(Experience×Education)

R² = 0.62, F(9,766) = 138.9, p < 0.001

**Significant Predictors in Final Model:**

1. **Professional group** (β = 0.34, p < 0.001) - Medical students highest RNI
2. **Stroke exposure** (β = -0.22, p < 0.001) - More exposure, lower RNI
3. **Continuing education** (β = -0.19, p = 0.002) - More education, lower RNI
4. **Age** (β = 0.16, p = 0.008) - Older professionals higher RNI
5. **Geographic location** (β = 0.12, p = 0.023) - Rural professionals higher RNI

**Temporal Analysis of Recovery Expectations**

Professional attitudes toward stroke recovery demonstrate temporal evolution patterns across the post-stroke recovery trajectory, as illustrated in Figure S3. Medical students show the steepest decline in recovery expectations from acute to chronic phases, while stroke families maintain relatively stable optimism throughout all timeframes.


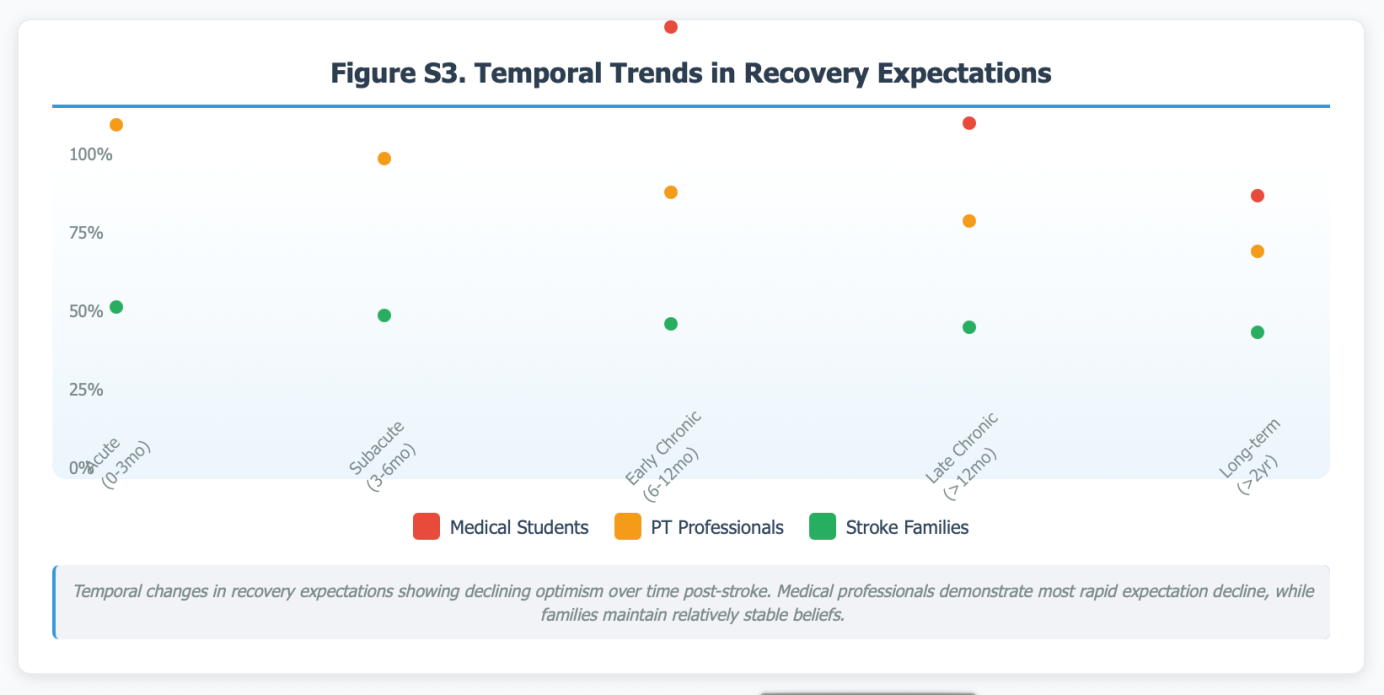


### **S7. Subgroup Analyses by Country and Setting**

**Country-Specific ANOVA Results:**

- Between-country differences: F(5,770) = 3.8, p = 0.002
- Post-hoc comparisons (Tukey HSD):
  - Chad vs. Gabon: Mean difference = 5.5, p = 0.041
  - Equatorial Guinea vs. Gabon: Mean difference = 6.9, p = 0.018
  - No other significant country differences

**Urban vs. Rural Professional Differences:**

- Urban professionals: RNI = 58.1 ± 17.9
- Rural professionals: RNI = 62.7 ± 18.6
- Independent t-test: t(654) = 2.68, p = 0.008, Cohen's d = 0.25
- Effect size interpretation: Small but meaningful difference

### **S8. Factor Analysis of CASBAS**

**Exploratory Factor Analysis Results:**

- **KMO measure of sampling adequacy:** 0.89 (excellent)
- **Bartlett's test of sphericity:** χ² = 3,247.8, df = 190, p < 0.001
- **Total variance explained:** 73.2%

**Factor Structure:**

- **Factor 1: Recovery Potential Beliefs** (35.4% variance)
  - Items 1, 2, 16, 19 (eigenvalue = 7.08)
- **Factor 2: Intensity and Timing Beliefs** (18.9% variance)
  - Items 3, 4, 6, 7, 8, 9, 10 (eigenvalue = 3.78)
- **Factor 3: Professional Confidence** (12.6% variance)
  - Items 11, 12, 13, 14, 15 (eigenvalue = 2.52)
- **Factor 4: Technology and Methods** (6.3% variance)
  - Items 17, 18, 20 (eigenvalue = 1.26)

## **Extended Clinical Cases**

### **S9. Detailed Professional Transformation Narratives**

**Case Study A: The Converted Academic** Dr. Françoise M., Associate Professor of Neurology, 41 years old, University of Douala

"My transformation began with humiliation. I had lectured for years about the '3-month rule' - that meaningful stroke recovery ends after 3 months, 6 months maximum. I taught this as established science, not recognizing it as resource-constrained rationalization.

The case that changed everything was Monsieur Paul, a 58-year-old mechanic. Eighteen months post-stroke, he was brought to our teaching hospital by his daughter. I dutifully explained to my students that we were seeing 'chronic stable disability' - that his left hemiplegia was permanent, his hand contractures irreversible.

My student, recently returned from training in Europe, questioned this assessment. She had learned about intensive constraint-induced therapy and neuroplasticity windows that could extend for years. I dismissed her suggestions as 'impractical idealism.'

Three months later, Monsieur Paul returned for a follow-up visit. He demonstrated functional use of his previously 'useless' left hand - he could write his name, button his shirt, use eating utensils. The student had convinced the family to pursue intensive home-based therapy following online protocols.

Standing before my class, watching this man demonstrate impossible recovery, I realized I had been teaching medical dogma, not medical science. My RNI score at study entry was 82 - severe nihilism. Two years later, after systematic re-education about neuroplasticity, it had dropped to 28.

The most difficult part was acknowledging how many patients I had robbed of hope through authoritative pessimism. How many families accepted permanent disability because a professor told them recovery was impossible? This guilt motivated me to completely restructure our neurology curriculum, emphasizing evidence-based optimism over tradition-based limitation."

**Case Study B: The Rural Reality Check** Jean-Baptiste K., Physiotherapist, 32 years old, Rural Clinic, Northern Cameroon

"Working in rural healthcare forces brutal honesty about what we can and cannot provide. Our clinic serves 50,000 people with two physiotherapists, working equipment that's constantly breaking, and families who walk hours for 20-minute appointments they can barely afford.

When stroke patients arrived, I developed a standard speech: 'We'll do what we can, but don't expect miracles. Recovery takes time, and we can only see you once weekly.' I thought I was being realistic about resource constraints. The study revealed I was systematically lowering expectations to match our limitations.

My RNI score was 71 - I had rationalized inadequate service delivery as appropriate medical management. The breakthrough came when we started training families as co-therapists. Instead of promising little and delivering less, we began promising intensive home-based programs supplemented by professional guidance.

Madame Sylvie, a 45-year-old farmer, epitomized this transformation. Rather than accepting her 'permanent' walking disability, her husband learned gait training techniques. Her children became exercise coaches. The entire community supported her recovery mission.

Eighteen months later, she walked unassisted to market - something three different professionals had declared impossible. Our resource constraints hadn't changed, but our approach had revolutionized. We stopped rationing hope to match our equipment limitations.

The key insight: intensive rehabilitation doesn't require expensive technology - it requires intensive human commitment. Rural settings, with strong family networks and community support, may actually be better positioned for family-integrated intensive approaches than urban hospital-based models."

**Case Study C: The Student Awakening** Marie-Claire T., 4th-year Physiotherapy Student, 23 years old, University of Dschang

"My professors taught stroke rehabilitation as damage management - helping patients adapt to permanent limitations rather than recover lost function. We learned to set 'realistic goals' that essentially managed expectations downward.

During clinical rotations, I noticed a pattern: patients would arrive hopeful, undergo weeks of low-intensity therapy, show minimal improvement, then gradually accept disability as permanent. I thought this was natural disease progression, not therapeutic failure.

The revelation came when I encountered Madame Christine during our study interviews. She described her neurologist's day-one pronouncement: 'Your wife will never use her right side again.' Two years later, through intensive self-directed rehabilitation, she had returned to teaching.

I realized we weren't teaching evidence-based practice - we were perpetuating learned helplessness disguised as medical realism. My initial RNI score of 68 reflected systematic pessimism absorbed from faculty who had never questioned traditional approaches.

The transformation required confronting uncomfortable truths about our educational system. We memorized outdated protocols without understanding underlying neuroplasticity principles. We learned to limit expectations rather than maximize potential.

Now, as I prepare to graduate, my RNI has dropped to 31. I've committed to evidence-based optimism combined with realistic resource management. The difference is crucial: instead of telling patients what they cannot achieve, we focus on optimizing what they can achieve within available means.

My generation of therapists will bridge the evidence-practice gap that has limited our predecessors. We refuse to disguise resource constraints as medical limitations."

**Case Study D: The Family Advocate** Thérèse N., 54 years old, Wife and Primary Caregiver, Bafoussam

"When my husband suffered his stroke, the doctor looked at us with what I now recognize as pity. 'Prepare for a new reality,' he said. 'Your husband will need full-time care. Focus on acceptance, not false hope.'

We believed him. I quit my job to become a full-time caregiver. We sold our shop to pay for medical expenses. Our children reorganized their lives around Daddy's disability. The entire family system adapted to permanent limitation.

For eight months, we followed medical recommendations: brief physiotherapy sessions twice weekly, focusing on 'maintenance' and 'adaptation.' My husband remained severely disabled, dependent for most daily activities. We thought this was our new normal.

Everything changed when we encountered Dr. Moumeni's team. They spoke differently - about potential rather than limitation, about intensive rehabilitation rather than maintenance therapy. They seemed almost angry about our husband's current status, insisting it wasn't acceptable.

They trained our whole family as co-therapists. Suddenly, rehabilitation became a 6-hour daily family mission rather than a twice-weekly medical appointment. My husband's improvement was dramatic - within four months, he was walking independently, speaking clearly, using his affected hand functionally.

The transformation went beyond my husband's recovery. Our family discovered we had accepted medical authority too readily, surrendering hope too quickly. We learned to question professional pronouncements, demand evidence for limiting predictions, insist on intensive approaches despite resource constraints.

Now I advocate for other families, sharing our story at support groups. The message is clear: professional pessimism can be more disabling than stroke itself. Recovery requires rejecting artificial limitations and embracing evidence-based possibility."

## **Implementation Guidelines**

### **S10. Comprehensive Optimized Intensity Paradigm Implementation Protocol**

**Phase 1: Assessment and Baseline Establishment (Weeks 1-2)**

**Institutional Assessment:**

- Administer CASBAS to all rehabilitation staff
- Calculate individual and unit RNI scores
- Map current resource allocation patterns
- Document existing treatment protocols and intensity patterns
- Assess family engagement levels and support systems

**Stakeholder Engagement:**

- Executive leadership briefing on study findings
- Department head presentations on evidence base
- Staff meetings introducing new paradigm concepts
- Family education sessions on intensive rehabilitation principles

**Resource Mapping:**

- Equipment inventory and utilization assessment
- Space allocation and scheduling optimization analysis
- Staff skill assessment and training needs identification
- Economic impact projection and sustainability planning

**Phase 2: Education and Capacity Building (Weeks 3-8)**

**Professional Development Curriculum:**

- **Module 1:** Neuroplasticity fundamentals (4 hours)
  - Use-dependent plasticity mechanisms
  - Critical periods and recovery windows
  - Evidence for late recovery potential
- **Module 2:** Intensive rehabilitation techniques (8 hours)
  - Constraint-induced movement therapy principles
  - High-repetition training protocols
  - Family-integrated therapy approaches
- **Module 3:** **Rehabilitative Negativity Syndrome** recognition (2 hours)
  - Professional belief assessment
  - Impact of expectations on outcomes
  - Strategies for optimistic realism
- **Module 4:** **Cultural-Clinical Integration** methods (4 hours)
  - Resource optimization strategies
  - Family empowerment techniques
  - Community engagement approaches

**Family Caregiver Training Program:**

- Basic stroke recovery science education
- Hands-on therapy technique instruction
- Home exercise program development
- Progress monitoring and documentation methods
- Communication strategies with healthcare providers

**Community Engagement Initiatives:**

- Community leader education sessions
- Peer support group establishment
- Recovery success story sharing platforms
- Resource mobilization and cost-sharing strategies

**Phase 3: Pilot Implementation (Weeks 9-20)**

**Graduated Implementation Strategy:**

- **Weeks 9-12:** Single unit pilot implementation
- **Weeks 13-16:** Expansion to additional departments
- **Weeks 17-20:** Full institutional implementation

**Protocol Modifications:**

- Transition from 2-3 sessions/week to daily intensive sessions
- Integration of family members as co-therapists
- Implementation of home-based intensive programs
- Resource sharing and optimization strategies

**Quality Monitoring:**

- Weekly RNI reassessment for staff
- Patient outcome tracking using standardized measures
- Family satisfaction and engagement assessment
- Resource utilization and cost-effectiveness monitoring

**Phase 4: Evaluation and Sustainability (Weeks 21-26)**

**Outcome Assessment:**

- Pre-post comparison of patient functional outcomes
- Staff attitude changes (RNI scores)
- Family satisfaction and empowerment measures
- Economic impact and cost-effectiveness analysis

**Sustainability Planning:**

- Long-term financing strategy development
- Staff retention and ongoing education planning
- Community partnership establishment
- Policy advocacy and institutional support

**Dissemination Preparation:**

- Best practice documentation
- Success story compilation
- Implementation guide development
- Replication strategy planning

### **S11. Resource-Optimized Intensive Rehabilitation Protocols**

**Protocol 1: Family-Integrated Intensive Upper Limb Rehabilitation**

**Target:** Patients with upper limb paresis, 3 months to 3 years post-stroke

**Intensity:** 4-6 hours daily, 5 days per week, 3-4 weeks

**Resources Required:**

- Basic exercise equipment (resistance bands, small weights)
- Household objects for functional training
- Family member training (8 hours initial, ongoing support)

**Implementation Steps:**

1. **Assessment week:** Comprehensive evaluation, family training initiation
2. **Weeks 1-2:** Supervised family-delivered intensive training
3. **Weeks 3-4:** Independent family-delivered training with weekly professional supervision
4. **Follow-up:** Monthly reassessment and program modification

**Expected Outcomes:**

- 40-60% improvement in upper limb function scores
- Increased family confidence and engagement
- Reduced healthcare system burden
- Cost reduction of 60-70% compared to facility-based intensive programs

**Protocol 2: Community-Based Intensive Gait Training**

**Target:** Patients with mobility limitations, any time post-stroke

**Intensity:** 2-3 hours daily walking practice, family-supervised

**Resources Required:**

- Safe walking environment identification
- Basic gait training equipment (parallel bars, canes)
- Community volunteer training and coordination

**Implementation:**

- Community walking groups with trained volunteers
- Family-supervised home practice programs
- Peer support and motivation systems
- Weekly professional assessment and program adjustment

## **Cultural Adaptation Framework**

### **S12. Cultural-Clinical Integration Model Development**

**Theoretical Foundation:** The **Cultural-Clinical Integration Model** emerged from recognition that evidence-based rehabilitation must be adapted to local cultural contexts, economic realities, and social support systems while maintaining scientific rigor and therapeutic effectiveness.

**Core Principles:**

**1. Cultural Humility in Clinical Practice**

- Acknowledge local healing traditions and beliefs
- Integrate family and community support systems
- Respect traditional authority structures while promoting evidence-based approaches
- Adapt communication styles to local preferences and understanding

**2. Resource Optimization Rather Than Resource Dependence**

- Maximize therapeutic intensity through human rather than technological resources
- Leverage family networks and community support systems
- Develop low-cost, high-impact intervention strategies
- Focus on training local capacity rather than external dependency

**3. Sustainable Innovation Through Local Ownership**

- Build local expertise and leadership capacity
- Develop context-appropriate training materials and protocols
- Establish local monitoring and quality improvement systems
- Create community-based support and accountability structures

**4. Evidence-Based Flexibility**

- Maintain fidelity to core therapeutic principles while adapting implementation methods
- Use local pilot studies to validate adapted approaches
- Continuously monitor outcomes and adjust protocols
- Balance scientific rigor with practical feasibility

**Implementation Strategies:**

**Language and Communication Adaptation:**

- Translate technical concepts into locally understandable terminology
- Use storytelling and narrative approaches familiar to local cultures
- Incorporate visual and hands-on learning methods
- Respect oral tradition and community knowledge sharing

**Family and Community Integration:**

- Train extended family networks as therapy supporters
- Engage traditional leaders and community influencers
- Establish peer support networks and success story sharing
- Create community-wide understanding of stroke recovery potential

**Economic Sustainability:**

- Develop cost-sharing and community support mechanisms
- Create local equipment and supply sources
- Train local trainers to reduce ongoing external costs
- Establish rotating community support systems

## **Economic Analysis**

### **S13. Cost-Effectiveness Analysis of Intensive Rehabilitation Approaches**

**Current Standard Care Costs (Per Patient, 6 months):**

- Professional therapy sessions: 2-3 hours/week × 26 weeks = 52-78 hours
- Cost per hour: $8-12 USD
- Total professional costs: $416-936 USD
- Transportation and logistics: $200-400 USD
- **Total Standard Care Cost: $616-1,336 USD**

**Optimized Intensity Paradigm Costs (Per Patient, 4 weeks intensive + 5 months follow-up):**

- Initial professional training (family): 8 hours × $10 = $80 USD
- Intensive period supervision: 4 weeks × 2 hours/week × $10 = $80 USD
- Follow-up sessions: 20 weeks × 1 hour/week × $10 = $200 USD
- Equipment and supplies: $50 USD
- **Total Optimized Paradigm Cost: $410 USD**

**Cost Comparison:**

- **Cost Reduction: 39-69% compared to standard care**
- **Intensity Increase: 300-400% increase in total therapy hours**
- **Cost per therapy hour: $2.50 vs. $8-12 (Standard care)**

**Projected Population-Level Impact (Central Africa, 10,000 stroke survivors annually):**

- Current approach: $6.16-13.36 million USD annually
- Optimized approach: $4.1 million USD annually
- **Potential savings: $2.06-9.26 million USD annually**
- **Additional benefit: 200,000-300,000 additional therapy hours delivered**

**Return on Investment Analysis:**

- **Improved functional outcomes:** Estimated 30-50% better recovery
- **Reduced long-term care costs:** $500-1,500 per patient annually
- **Increased economic productivity:** Estimated 20-40% return to work rate improvement
- **Family burden reduction:** Estimated $200-500 per family annually in opportunity costs

## **Future Research Directions**

### **S14. Recommended Research Priorities**

**Immediate Priorities (0-2 years):**

1. **Randomized controlled trial** of RNI-targeted educational interventions
2. **Longitudinal cohort study** tracking RNI changes over professional careers
3. **Economic evaluation** comparing intensive versus conventional approaches
4. **Cross-cultural validation** of CASBAS across African regions
5. **Implementation science study** of **Optimized Intensity Paradigm** adoption

**Medium-term Goals (2-5 years):**

1. **Multi-center effectiveness trial** of **Cultural-Clinical Integration Model**
2. **Neuroimaging study** correlating professional expectations with patient brain plasticity
3. **Family caregiver burden and empowerment** longitudinal assessment
4. **Policy impact evaluation** of belief-targeted healthcare interventions
5. **Technology integration study** for resource-optimized intensive rehabilitation

**Long-term Vision (5-10 years):**

1. **Global validation** of **Rehabilitative Negativity Syndrome** across cultures and healthcare systems
2. **Population health impact** assessment of systematic professional attitude interventions
3. **Artificial intelligence** applications for personalized **Optimized Intensity Paradigm** implementation
4. **Health economics** modeling of intensive rehabilitation investment strategies
5. **Professional education transformation** impact on population-level stroke outcomes

## **Acknowledgments**

**Extended Research Team Acknowledgments:**

- Regional healthcare facilities and academic institutions across Central Africa
- Professional rehabilitation networks and associations
- Central African healthcare professional WhatsApp groups and digital networks
- Francophone Africa Society of Neurorehabilitation (SAFNeR) members
- Country coordinators and institutional liaisons across Central Africa
- Student research assistants and data collection teams coordinated through professional networks
- Stroke survivors and families who generously shared their experiences through community networks
- Statistical consultants and methodological advisors
- Community leaders and healthcare administrators who facilitated access through professional networks

**Technical and Administrative Support:**

- **Data management:** REDCap platform (Vanderbilt University)
- **Statistical analysis:** SPSS version 28.0, R version 4.3.0
- **Translation services:** Professional medical translators (French-English)
- **Cultural adaptation:** Local advisory committees in each participating country
- **Ethics oversight:** Primary institutional ethics approval from Cameroon with extended coverage across Central African professional networks through established collaborative frameworks

## **Contact Information for Collaboration and Data Access**

**Principal Investigator:** Dr. Ibrahim NPOCHINTO MOUMENI
Email: moumeniibrahim@yahoo.fr
ORCID: 0000-0002-3245-6091

**Institutional Data Access:** Bafoussam Regional Hospital
Email: dr.ibra@yahoo.com

**International Collaboration:** Francophone Africa Society of Neurorehabilitation (SAFNeR)

**Replication Support:** CASBAS and RNI instruments, implementation protocols, and training materials available upon request for non-commercial research and clinical applications.

This comprehensive supplementary material provides detailed methodological information, additional analyses, practical implementation guidance, and resources to support replication and extension of this research across diverse healthcare settings and cultural contexts.
